# Trends in heart failure-related mortality among breast cancer patients in the United States from 1999 to 2024

**DOI:** 10.1016/j.ajpc.2026.101680

**Published:** 2026-05-25

**Authors:** Vikash Jaiswal, Danisha Kumar, Kriti Kalra, Fakhar Latif, Yusra Minahil Nasir, Anupam Halder, Maciej Banach, Garima Sharma, Gregg C. Fonarow, Ana Barac

**Affiliations:** a Endeavor Center for Cardiovascular Intervention Outcomes Research and Evaluation (ECCORE), Section of Interventional Cardiology, Endeavor Health Cardiovascular Institute, Glenview, IL 60201, USA; b University of Chicago Pritzker School of Medicine, Chicago, IL, United States; c Department of Medicine, Dow University of Health Sciences, Karachi, Pakistan; d Department of Cardiology, MedStar Washington Hospital Center, WD, United States; e Department of Internal Medicine, University of Oklahoma Health Science Center, Oklahoma City, OK, United States; f Columbia University Division of Cardiology at Mt. Sinai Medical Centre, Miami, United States; g Ciccarone Center for the Prevention of Cardiovascular Diseases, Johns Hopkins Medicine, Baltimore, Maryland, USA; h Inova Schar Heart and Vascular, Inova Fairfax Medical Campus, Falls Church, VA, USA; i Ahmanson-UCLA Cardiomyopathy Center, Ronald Reagan UCLA Medical Center, Los Angeles, CA, USA; j Georgetown University, WA DC, USA; k Faculty of Medicine, The John Paul II Catholic University of Lublin, Lublin, Poland

**Keywords:** Breast cancer, Heart failure, Mortality, Cardio-oncology

## Abstract

**Background:**

There is a paucity of data among women with breast cancer and heart failure (HF)-related mortality trends based on race, ethnicity, and geographic regions in the United States.

**Objective:**

To investigate the trends in HF-related mortality among breast cancer patients aged ≥25 years.

**Methods:**

This retrospective analysis utilized mortality data from the US Centers for Disease Control and Prevention (CDC) Wide-ranging Online Data for Epidemiologic Research (WONDER) database from 1999 to 2024. We analyzed temporal trends in the HF-related mortality in breast cancer patients aged ≥25 years. Crude (CMRs) and age-adjusted mortality rates (AAMRs) per 100,000 people with the associated annual percentage change (APC) and 95% confidence intervals (CIs) were calculated. Joinpoint regression was used to analyze trends across the overall sample and various demographic and geographic subgroups.

**Results:**

Between 1999 and 2024, 8398 death certificates identified HF as the primary cause and breast cancer as a contributory cause, resulting in an AAMR of 0.139 per 100,000. The overall AAMR significantly declined from 1999 to 2014 (APC: -5.443%, 95% CI: -6.436 to -4.440), followed by a significant increase from 2014 to 2024 (APC: 6.299%; 95% CI: 4.475 to 8.155). Racial disparities were noted, with NH Black patients exhibiting the highest AAMR of 0.176 (95% CI: 0.121 - 0.248). Geographical differences were also observed, with Mississippi having the highest AAMR of 0.324 (95% CI: 0.270 - 0.379) and Florida having the lowest AAMR of 0.053 (95% CI: 0.045 - 0.061). Additionally, from 1999 to 2020, nonmetropolitan areas consistently showed higher AAMRs than metropolitan areas with rates of 0.159 (95% CI: 0.151 - 0.168) and 0.119 (95% CI: 0.116 - 0.123), respectively. Among the age groups, the highest CMR was observed in patients aged ≥85 years (CMR: 4.859).

**Conclusion:**

The persistent rise in HF-related mortality, especially pronounced among NH Black patients and in specific geographic locales, underscores the need for enhanced collaborative efforts across oncology, cardiology, cardio-oncology, public health, and policy domains to lessen disparities and improve the overall health outcomes of breast cancer survivors.

## Nonstandard abbreviations and acronyms

AAMR: age‐adjusted mortality rates
CDC: Centers for Disease Control and Prevention
CMR: crude mortality rate

## 1 Introduction

Cancer remains one of the predominant causes of mortality worldwide, with breast cancer representing a significant cause of both mortality and morbidity. It is the most frequently diagnosed cancer and the second leading cause of cancer-related mortality in the United States (US) [1]. Although breast cancer mortality has shown a decline of 40% since 1975, the disparities among non-Hispanic (NH) black and NH white women persist [1,2]. The mortality rates are still consistently higher in NH Black women, with some studies reporting 41% higher breast cancer-related deaths among NH Black women. These disparities could be attributed to a multitude of factors, including factors that differentially impact women across various racial groups. Moreover, breast cancer survivors have a significantly increased risk of death due to cardiovascular disease (CVD) [3]. There are many factors influencing this, including higher inflammation and oxidative stress promoting atherosclerosis progression, side effects of anti-cancer drugs, and a cancer-centric treatment approach that lacks a comprehensive cardiovascular health perspective. Recent literature suggests that cancer patients receiving chemotherapy have demonstrated 35% higher risk of developing congestive heart failure (HF) [4]. Additionally, according to a study, among 1059,048 breast cancer patients diagnosed from 1992 to 2014, 4.6% died from heart disease, highlighting an association between CVD and breast cancer [5]. Increased awareness regarding the cardiotoxicity associated with chemotherapy has resulted in changes in breast cancer treatment regimens, such as reduced usage of anthracycline and modification of radiation therapy. However, there is a gap in knowledge about the effect of these changes on cardiovascular outcomes, including HF.

In this study, we utilized the CDC WONDER database to investigate HF-related mortality trends over 25 years in patients with a history of breast cancer in the United States. We also analyzed demographic and geographical variations in HF-related mortality among this clinically significant population.

## 2 Methods

### 2.1 Study setting and population

We conducted a descriptive analysis of death certificate data sourced from the CDC WONDER (Wide-Ranging Online Data for Epidemiologic Research), a publicly available database [6]. CDC WONDER offers mortality data for US residents and patients across all US counties from 1999 to 2024. Each entry in the database presents one primary cause of death along with up to 20 additional factors contributing to mortality, alongside demographic information like age, sex, and race/ethnicity. The primary cause of death refers to the disease directly leading to the demise while contributing factors encompass other conditions associated with the fatal outcome. Both primary and contributing causes of death are classified using the International Classification of Diseases, Tenth Revision (ICD-10). The database allows retrieval of death counts, crude mortality rates (CMRs), and age-adjusted mortality rates (AAMRs) categorized by primary cause, residency, age, race, sex, and year of death.

We included individuals aged ≥25 years in whom breast cancer (C50) was listed as a contributing cause and HF (I11.0, I13.0, I13.2, I50.x) as the primary (underlying) cause of death. The Multiple Cause-of-Death Public Use record death certificates were studied to select HF-related deaths in breast cancer patients, which were identified as those with HF reported as underlying cause of death on the death certification while breast cancer as contributing cause of death reported anywhere on the death certification. Our study did not require institutional review board approval, as we exclusively utilized publicly available data. We followed the Strengthening the Reporting of Observational Studies in Epidemiology (STROBE) guidelines [7].

### 2.2 Data abstraction

In the CDC WONDER database, data abstraction was already performed, and we collected mortality information stratified by population size, year, age categories, census region, states, location of death, and metropolitan/nonmetropolitan status. Individuals aged ≥25 years were included. Race or ethnicity was stratified into NH white, NH black or African American, Hispanic or Latino, NH American Indian or Alaska Native, and NH Asian or Pacific Islander. These race and ethnicity categories have previously been used within analyses from the Centers for Disease Control and Prevention Wide-Ranging Online Data for Epidemiological Research database and rely on reported data on death certificates [6]. Place of death encompassed hospitals (inpatient, outpatient, death on arrival, or status unknown), the decedent’s home, hospice, nursing homes, or long-term care facilities. Census regions included the Midwest, South, West, and Northeast, according to the definitions of the U.S. Census Bureau [8]. The National Center for Health Statistics Urban-Rural Classification Scheme was used to assess the population by metropolitan (large metropolitan area [population ≥ 1 million], medium/small metropolitan area [population 50,000–999,999]) and nonmetropolitan (population <50,000) areas per the 2013 U.S. census classification [8].

### 2.3 Statistical analysis

CMRs and AAMRs per 100,000 individuals were calculated for the period spanning 1999 to 2024. CMRs were derived by dividing the total number of HF-related deaths in breast cancer patients by the corresponding U.S. population for each year. AAMRs were calculated by standardizing the crude rates against the U.S. population in the year 2000, as described by the National Center for Health Statistics [9]. This standardization allows for comparison across years while accounting for age distribution changes in the population. These demographic data, including sex, ethnicity, geographical location (state), and urban or rural residency status, were collected from the Centers for Disease Control and Prevention (CDC) database. AAMR was used for all analyses except those stratified by the age categories, where CMR was used. We utilized the National Cancer Institute’s JoinPoint Regression Program (Version: 5.0.2) [10] to evaluate the mortality fluctuations over time. This involved fitting log-linear regression models to derive the annual percent change (APC) in AAMRs. To determine whether APCs were statistically different from zero (indicating a significant trend), a 2-tailed *t*-test was performed. We set the threshold for statistical significance at a P value of < 0.05.

We excluded certain age groups and race populations from our trend analysis due to unreliable or suppressed data in the CDC Wonder database. The specific age groups of 25–34, 35–44, 45–54, and 55–64 were not included in the trend analysis because the CDC suppressed or flagged the data as unreliable based on their policy. According to CDC guidelines, statistics are suppressed when representing fewer than ten deaths, and rates are considered unreliable when the death count is fewer than 20. Therefore, we restricted our analysis to age groups where reliable data was available. Similarly, for race and ethnicity, detailed mortality reports for specific populations, including Hispanic or Latino, Asian or Pacific Islander, and American Indian or Alaska Native, were suppressed or unreliable. As a result, we excluded these populations from our stratified analysis and focused on populations where reliable data was available. We also limited our analysis for nonmetropolitan and metropolitan areas till 2020 because essential characteristics of mortality data such as AAMRs and population, are suppressed or unreliable in the CDC database for the years 2021–2024.

## 3 Results

Between 1999 and 2024, a total of 8398 death certificates with HF-related mortality as a primary cause of death and BC as a contributory cause of mortality were identified. This corresponds to an overall AAMR of 0.139 per 100,000. The crude HF mortality (CMR) in patients with BC increased from 0.196 to 0.210 between 1999 and 2024.

### 3.1 Annual trends for HF-related AAMR in breast cancer patients

**Fig. 1:**
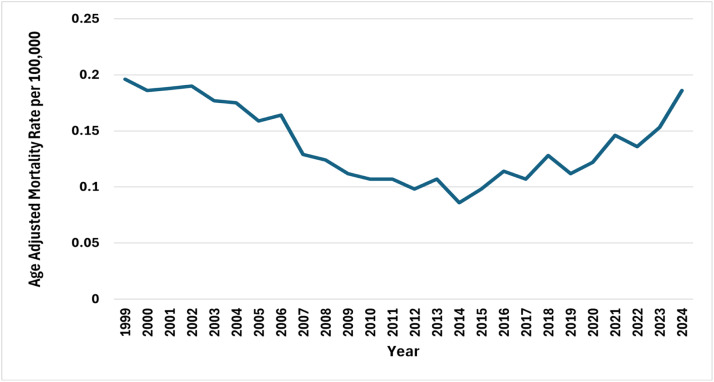
Overall Heart Failure related Age Adjusted Mortality rates (AAMRs) per 100,000 in Breast Cancer Patients in the United States, 1999 to 2024.

AAMR for HF-related deaths in adults was 0.196 in 1999 and 0.186 per 100,000 in 2024. The overall AAMR significantly declined from 1999 to 2014 (APC: −5.443%; 95% CI: −6.436 to −4.440), followed by a significant increase from 2014 to 2024 (APC: 6.299%; 95% CI: 4.475 to 8.155) (Fig. 1 and **S1)** (**Table S1**).

### 3.2 HF-related AAMR in breast cancer patients stratified by race/ethnicity

When stratified by race/ethnicity, AAMRs were highest among NH Black or African American patients followed by NH White, Hispanic or Latino, NH Asian or Pacific Islander and NH American Indian or Alaska Native populations (overall AAMR NH Black or African American: 0.176, 95% CI: 0.121 - 0.248; NH White: 0.140, 95% CI: 0.123 - 0.157; Hispanic or Latino: 0.072, 95% CI: 0.051 - 0.100; NH Asian or Pacific Islander: 0.050, 95% CI: 0.038 - 0.065; NH American Indian or Alaska Native: Unreliable, 95% CI: 0.034 - 0.104). Detailed reports on race populations such as Hispanic or Latino, NH Asian or Pacific Islander, NH American Indian or Alaska Native are suppressed or unreliable in the CDC database, and therefore, mortality trends in these race populations cannot be reliably assessed. The AAMR for NH Black or African American population non-significantly decreased from 1999 to 2024 (AAMR in 1999: 0.225; AAMR in 2024: 0.193; APC: −0.370; 95% CI: −1.521 to 0.803). The AAMRs for NH White exhibited a significant decrease from 1999 to 2014 (AAMR in 1999: 0.196; AAMR in 2014: 0.088; APC: −5.274; 95% CI: −6.178 to −4.360), followed by a steep significant incline from 2014 to 2024 (AAMR in 2014: 0.088; AAMR in 2024: 0.178; APC: 6.047; 95% CI: 4.246 to 7.880) (Figs. 2a, 2b, 2c, **and Figure S2).**

**Fig. 2a:**
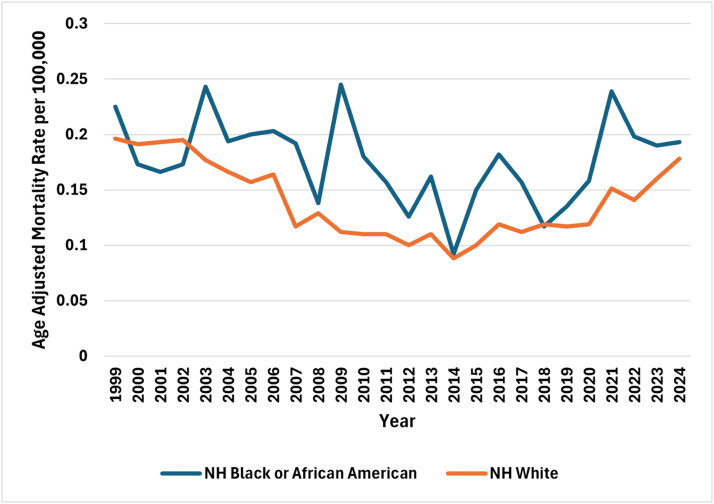
Trends in Heart Failure related AAMR among Breast Cancer patients stratified by race in the United States, 1999–2024.* *Detailed reports on race populations such as Hispanic or Latino, Asian or Pacific Islander, American Indian or Alaska Native are suppressed or unreliable in the CDC database and therefore have been excluded from this graph.

**Fig. 2b:**
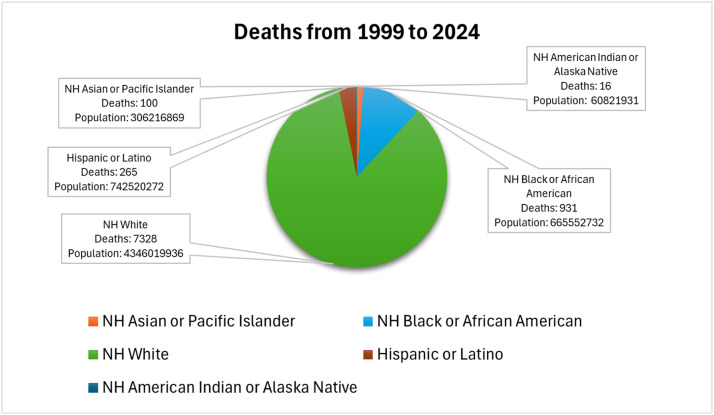
Heart Failure related deaths among Breast Cancer Patients stratified by race in the United States, 1999–2024.

**Fig. 2c:**
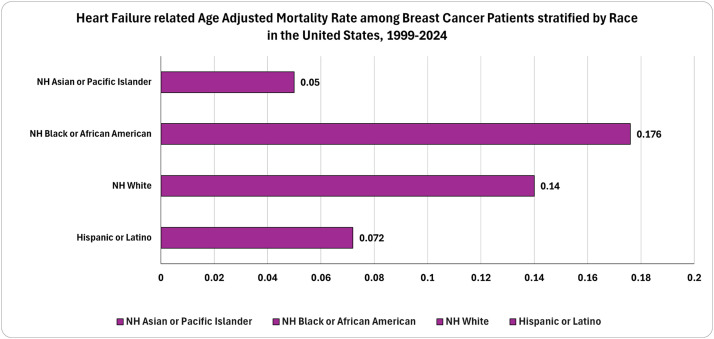
Heart Failure related AAMRs among Breast Cancer Patients stratified by race in the United States, 1999–2024.

During the study period, patients of Hispanic origin accounted for 3.16% (n = 265) of the HF-related mortality with BC as a contributing factor, whereas NH patients accounted for 96.83% (n = 8112) mortality (**Table S2).**

### 3.3 HF-related AAMR in breast cancer patients stratified by geographic region

**States:** From 1999 to 2020, a significant difference in AAMR was observed in different states, with the AAMRs ranging from 0.053 (95% CI: 0.045 - 0.061) in Florida to 0.324 (95% CI: 0.270 - 0.379) in Mississippi. States that fell into the top 90th percentile were Mississippi, Nebraska, Oklahoma, Colorado, Oregon, Wisconsin, and Minnesota, which had approximately thrice the AAMRs or more compared with states that fell into the lower 10th percentile, namely Florida, Arizona, New Mexico, Tennessee, Rhode Island, Maine, and Louisiana (Fig. 3a, Fig. 3b) (**Table S3).**

**Fig. 3a:**
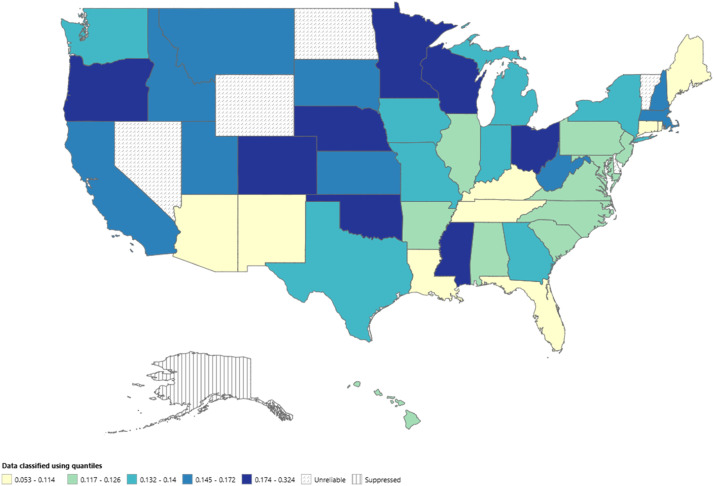
Heart Failure related AAMRs among Breast Cancer Patients across 50 states in the United States, 1999–2020* *Detailed reports on mortality data of the states, including AAMRs and population, are suppressed or unreliable in the CDC database for the years 2021–2024, and therefore have been excluded from this map.

**Fig. 3b:**
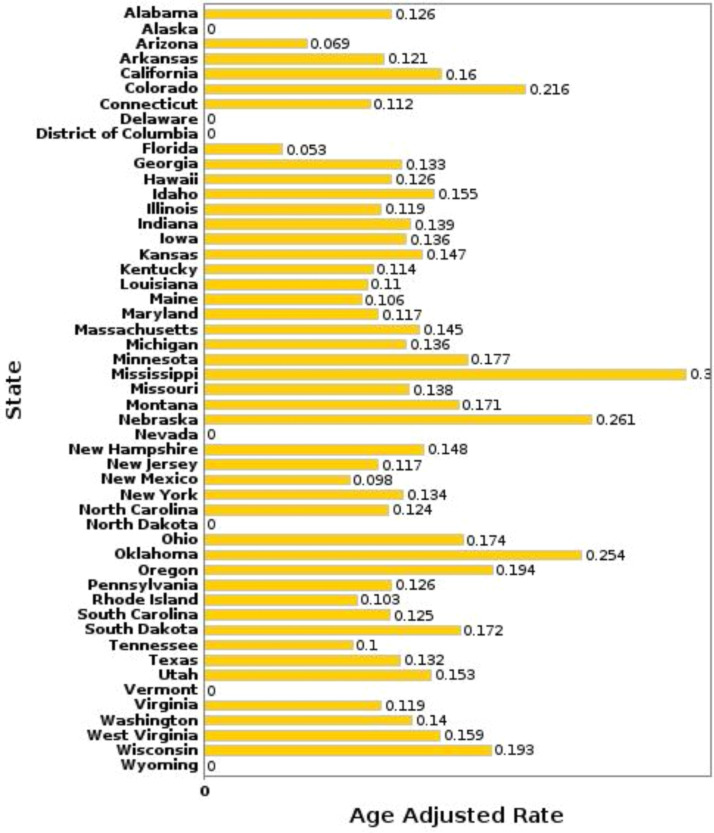
Heart Failure related AAMRs among Breast Cancer Patients across 50 states in the United States, 1999–2020* *Detailed reports on mortality data of the states, including AAMRs and population, are suppressed or unreliable in the CDC database for the years 2021–2024, and therefore have been excluded from this chart.

**Fig. 4:**
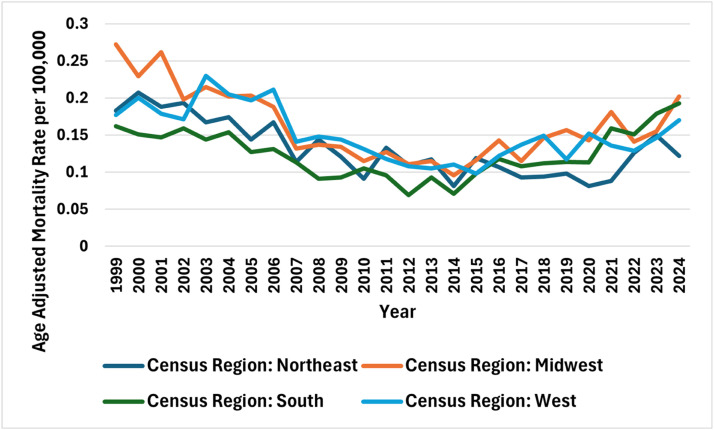
Trends in Heart Failure related AAMR among Breast Cancer patients stratified by census regions in the United States, 1999–2024.

**Census Regions:** On average, over the course of the study period the highest mortality was observed in the Midwestern (AAMR: 0.163; 95% CI: 0.129 - 0.202), followed by the Western region (AAMR: 0.151; 95% CI: 0.117 - 0.191), Northeastern (AAMR: 0.131; 95% CI: 0.100 - 0.169), and Southern (AAMR: 0.125; 95% CI: 0.101 - 0.152) regions. The AAMR in the Midwestern region had a significant decline from 1999 to 2013 (APC: −6.510; 95% CI: −7.936 to −5.061), followed by a significant rise from 2013 to 2024 (APC: 5.280; 95% CI: 2.953 to 7.656). The AAMR in the Western region non-significantly increased from 1999 to 2004 (APC: 4.150; 95% CI: −3.088 to 11.921), followed by a significant decline from 2004 to 2012 (APC: −8.570; 95% CI: −12.555 to −4.400), after which there has been a significant increase from 2012 to 2024 (APC: 3.370; 95% CI: 1.415 to 5.356). The Northeastern region had a significant decline in AAMR from 1999 to 2020 (APC: −3.960; 95% CI: −4.891 to −3.012), followed by a sharp and significant rise from 2020 to 2024 (APC: 14.64; 95% CI: 0.46 to 39.8). The AAMR in the Southern region had a significant decline from 1999 to 2014 (APC: −4.820; 95% CI: −6.173 to −3.442), followed by a sharp and significant rise from 2014 to 2024 (APC: 8.810; 95% CI: 6.500 to 11.162) (Figs. 4 **and Figure S3**) (**Table S4)**.

**Fig. 5:**
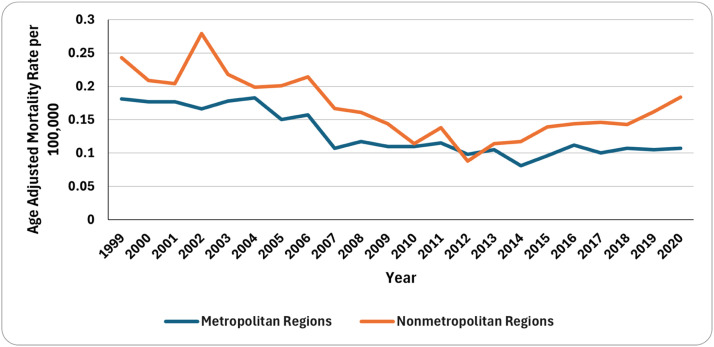
Trends in Heart Failure related AAMR among Breast Cancer patients stratified by urbanization in the United States, 1999–2020.* *Detailed reports on mortality data of the metropolitan and nonmetropolitan regions, including AAMRs and population, are suppressed or unreliable in the CDC database for the years 2021–2024, and therefore have been excluded from this graph.

**Rural-Urban Classification**: Detailed reports on mortality data of the metropolitan and nonmetropolitan areas, including AAMRs and total population, are suppressed or unreliable in the CDC database for the years 2021–2024, and therefore have been excluded from this trend analysis. From 1999 to 2020, nonmetropolitan areas had consistently higher HF-related AAMRs in BC patients than metropolitan areas, with overall AAMRs of 0.159 (95% CI: 0.151 - 0.168) and 0.119 (95% CI: 0.116 - 0.123), respectively. Furthermore, the AAMRs of nonmetropolitan areas exhibited a significant decrease from 1999 to 2013 (APC: −5.840; 95% CI: −7.673 to −3.960), which was then followed by a significant increase from 2013 to 2020 (APC: 6.261; 95% CI: 0.577 to 12.268). Similarly, AAMRs of metropolitan areas exhibited a significant decline from 1999 to 2014 (APC: −4.812; 95% CI: −5.908 to −3.704), which was then followed by a non-significant increase from 2014 to 2020 (APC: 2.700; 95% CI: −2.175 to 7.819) (Figs. 5 **and Figure S4**) (**Table S5**).

### 3.4 HF-related CMR in breast cancer patients stratified by ten-year age groups

During the study period, patients aged ≥85 years had the highest CMR (4.859), accounting for almost 58.43% (n = 4873). This is followed by patients between the ages 75–84 (CMR: 0.952), 65–74 years (CMR: 0.256), 55–64 years (CMR: 0.069), 45–54 years (CMR: 0.017), and 35–44 years (CMR: 0.005). Data for age group 25–34 were suppressed or unreliable in the CDC database and therefore were excluded (Fig. 6a, Fig. 6b) (**Table S6).**

**Fig. 6a:**
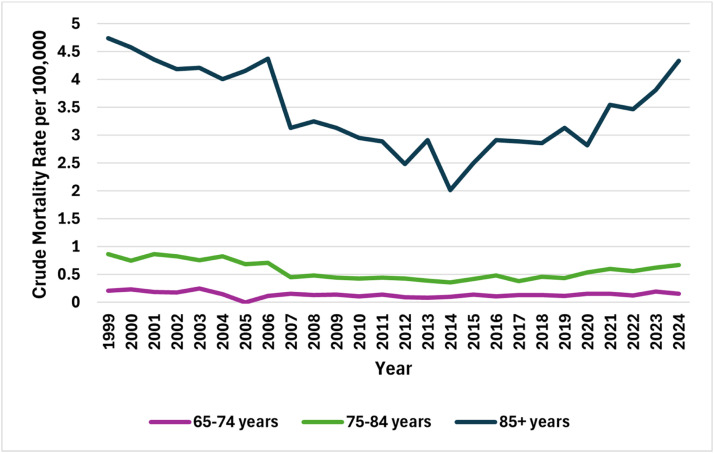
Trends in Heart Failure related CMR among Breast Cancer patients stratified by ten year age groups in the United States, 1999–2024.* *Trends on age groups such as 25–34, 35–44, 45–54, 55–64 are suppressed or unreliable in the CDC database and therefore have been excluded from this graph.

**Fig. 6b:**
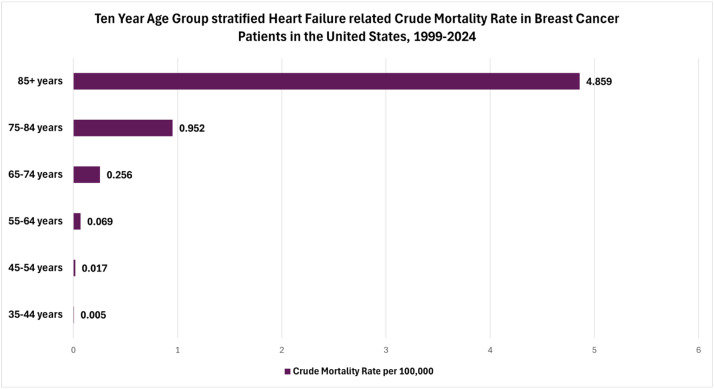
Heart Failure related CMRs among Breast Cancer patients stratified by ten year age groups in the United States, 1999–2024.

### 3.5 HF-related trends in breast cancer patients stratified by location of death

**Fig. 7:**
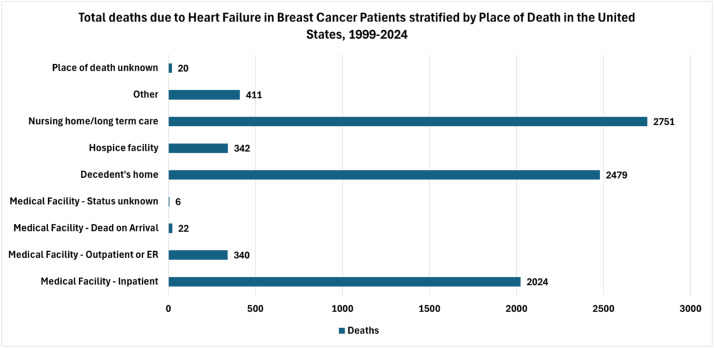
Total deaths due to Heart Failure in Breast Cancer patients stratified by Place of Death in the United States, 1999–2024.

Information for the location of death was available for 8395 deaths. Of these, 32.77% occurred in nursing homes/long-term care facilities, 29.53% occurred at decedent’s home, 24.11% occurred within inpatient medical facilities, 4.05% deaths occurred in outpatient medical facilities or ER, 0.07% in medical facilities (status unknown), 4.07% occurred in hospices, 4.90% occurred at places other than those listed, 0.24% occurred in unknown places, and 0.26% were considered dead on arrival at medical facilities (Fig. 7) (**Table S7).**

## 4 Discussion

In this nationwide, descriptive analysis of mortality data spanning 1999–2024, we observed a dynamic pattern in HF-related mortality among individuals in whom breast cancer was listed as a contributing condition on the death certificate. Mortality rates declined significantly through 2014 before rising through 2024. We also identified notable racial, geographic, and rural-urban disparities. Importantly, because this analysis relies on death certificate data, all findings represent observed associations and cannot be used to infer causality between breast cancer, its treatment, and fatal HF (**Central Illustration**).

### 4.1 Dynamic shifts in mortality rates

The observed biphasic trend, a significant AAMR decline from 1999 to 2014 (APC: −5.4%), followed by a significant rise through 2024 (APC: 6.3%), is consistent with broad temporal shifts in both HF and cancer epidemiology in the US over this period [11,12]. The post-2014 reversal may coincide with changes in breast cancer treatment practices, increasing breast cancer survivorship, and a rising background prevalence of HF and its shared risk factors, although this dataset is not designed to distinguish between these possibilities.

The post-2014 increase in HF-related mortality observed in this study is temporally consistent with several developments in the breast cancer treatment landscape [13]. Over the past decade, treatment protocols for all stages of breast cancer have incorporated novel targeted therapies alongside traditional chemotherapeutic agents, including Trastuzumab for HER2-positive disease [14,15], Pertuzumab in combination regimens [16,17], aromatase inhibitors in hormone receptor-positive disease [18], and poly(ADP-ribose) polymerase (PARP) inhibitors for triple-negative disease [19]. Each of these agents has been associated with cardiovascular effects in clinical trial and pharmacovigilance data, and their expanded use since the early 2010s coincides broadly with the inflection point observed in our trend data [[13], [14], [15], [16], [17], [18], [19]]. These temporal associations are hypothesis-generating and consistent with the possibility that widening use of cardiotoxic regimens contributes to the observed HF mortality signal; however, the absence of treatment exposure data in CDC WONDER prevents any direct attribution.

Furthermore, the concept of the 'common soil' phenomenon, which highlights shared risk factors between HF and cancer, suggests an overarching increase in these shared risk factors, further compounding the cardiovascular outcomes in breast cancer survivors [20]. In breast cancer patients, higher body mass index (BMI) is linked to a decline in left ventricular ejection fraction (LVEF) within three months of treatment initiation [21,22]. Anthracycline-associated cardiotoxicity has been found to be more pronounced in patients with higher BMI [23]. The French-CANTO study further showed a strong correlation between obesity and an elevated risk of cardiotoxicity [13]. The effects of metabolic diseases are not limited to HF with reduced Ejection Fraction (HFrEF) alone. The rising prevalence of obesity as well as metabolic syndrome over the past decade and its association with HF with preserved Ejection Fraction (HFpEF), including sex-specific effects, is associated with higher HF-associated mortality among breast cancer survivors [[24], [25], [26]]. Moreover, the rising incidence of HFpEF in post-systemic chemotherapy, as well as its limited treatment, might have further contributed to increased HF-related mortality in breast cancer survivors [27].

As the survivorship age for breast cancer patients has increased over the last decade, these individuals also face an increasing burden of traditional cardiovascular risk factors such as hypertension, diabetes, and dyslipidemia [28]. These risks may coincide with the effects noted in the common soil hypothesis or emerge as direct consequences of chemotherapy-induced cardiotoxicity [29]. Notably, HF-related mortality has risen in the US over the last decade, consistent with our observed mortality trends and providing a broader context for understanding these developments in breast cancer survivors [[30], [31], [32]].

### 4.2 Racial and geographic disparities

Our analysis reveals significant racial and geographic disparities in HF-related mortality among breast cancer survivors. Despite a modest reduction in mortality rates among NH Black women, from 0.225 in 1999 to 0.193 per 100,000 persons in 2024, disparities remain stark. These racial disparities were mirrored geographically, with states such as Mississippi demonstrating substantially higher AAMRs, potentially reflecting the intersection of racial composition, socioeconomic disadvantage, and healthcare access. This observation aligns with broader literature indicating that systemic inequities in healthcare access and quality persistently disadvantage non-White populations, particularly Black and Asian women, who experience significant delays and lower likelihood of receiving essential therapies such as hormone and immunotherapy [33,34].

Socioeconomic status significantly influences these outcomes, with poverty emerging as a critical factor exacerbating disparities in breast cancer incidence, survival, and mortality. Low-income women, especially from Black communities, are less likely to have regular healthcare providers, resulting in lower rates of mammography screening and a greater probability of late-stage diagnoses [34]. Additionally, these women often receive substandard treatment due to economic barriers and limited access to equipped healthcare facilities, further elevating their risk of mortality from breast cancer. These socioeconomic factors contribute to the observed disparities as much as, if not more than, racial differences alone, highlighting the intertwined impact of economic and racial inequalities on health outcomes [34]. Compounded with this is the known mortality disparity gap in NH Black patients [35]. Hence, the results of our study, although not surprising, continue to paint a dismal gap in care for racial and ethnic minorities.

Addressing these disparities requires targeted strategies that address racial inequalities and confront the socioeconomic determinants of health. Ensuring equitable access to comprehensive healthcare, improving infrastructure in underserved areas, and enhancing patient education are essential steps toward reducing these disparities and improving patient management across diverse populations.

### 4.3 Impact of age distribution on mortality trends

A critical finding that should anchor interpretation of aggregate trends is the age distribution of deaths. Patients aged ≥85 years accounted for 58.43% of all HF-related deaths among breast cancer patients, far exceeding other age groups. This is expected, as both HF and overall mortality rise steeply with age, independent of breast cancer history [1,5,11,31]. In this elderly population, breast cancer is often remote, and the temporal relationship between cancer treatment and HF-related death cannot be established from death certificate data [1].

Clinically, a more relevant population for potential treatment-related cardiotoxicity includes individuals aged 65–84 years, where cancer diagnosis and exposure to cardiotoxic therapies are more likely to be temporally proximate to death [3,13,15,23]. In these groups, CMRs were 0.256 and 0.952 per 100,000, respectively, and the post-2014 increase in overall AAMR may be most reflective of trends within this population. However, the CDC WONDER database does not capture cancer diagnosis timing or treatment exposure, precluding causal inference. Furthermore, younger women, particularly those aged 45–54, who are most likely to receive intensive anthracycline- and trastuzumab-based regimens and who represent a clinically critical population for cardiotoxicity monitoring, were largely excluded due to data suppression [3,13,15,23]. This suppression is a direct consequence of the rarity of fatal HF in this age group within the population-level surveillance framework [11]. Future analyses using SEER-Medicare linked data or the National Inpatient Sample (NIS) database will be better positioned to characterize HF events, including non-fatal HF hospitalizations and cardiotoxicity during active treatment, among younger breast cancer patients.

### 4.4 Clinical implications and future directions

#### 4.4.1 Enhancing early detection and advancing survivorship care

Current European Society of Cardiology (ESC) guidelines emphasize the necessity for early risk stratification of all patients initiating oncology treatment, recognizing that cardiovascular risk is dynamic and requires continuous monitoring and management of modifiable factors. Many cancer patients do not undergo routine cardiovascular risk monitoring, including e.g., measuring of the lipid profile, glucose, HbA1c, liver enzymes (the risk of SLD), kidney parameters, blood pressure monitoring, etc. and consultation, and the detailed evaluation is made while they are symptomatic. This process demands a collaborative, multidisciplinary approach involving both oncologists and cardiologists, particularly for patients at high risk or those who develop new onset HF during active cancer treatment [36]. Furthermore, integrating cardiologists into long-term survivorship care programs is crucial to enhance HF's early detection and management, potentially mitigating long-term adverse outcomes.

Looking forward, future research must focus on refining methods for early detection and precise risk stratification of HF in breast cancer survivors. This entails the development of sophisticated predictive models that seamlessly integrate clinical data with genetic and socio-demographic information to ensure comprehensive and personalized care. By incorporating advanced cardiovascular assessment techniques, such as detailed imaging and sensitive biomarkers, into routine survivorship care, clinicians can detect early signs of cardiotoxicity more effectively.

Moreover, there is a critical need to develop tailored intervention strategies that consider individual risk profiles, past treatment histories, and socio-demographic factors. These strategies should aim to treat existing conditions and proactively manage potential cardiovascular risks associated with cancer therapies. This comprehensive approach ensures that all survivors, regardless of their background or treatment regimen, receive optimal care that addresses their oncological and cardiovascular health needs.

#### 4.4.2 Cardio-protection strategies

The integration of cardio-protective measures into the treatment plans of breast cancer survivors, particularly those at elevated risk for HF, is becoming increasingly vital. As anticancer therapy continues to improve overall survival (OS) and progression-free survival (PFS), it becomes increasingly necessary to comprehensively monitor other risk factors and comorbidities in this growing population of long-term survivors. Early and aggressive risk profiling and stratification at the onset of breast cancer therapy can significantly enhance outcomes. This approach is supported by emerging evidence suggesting that neurohormonal blocking agents can offer substantial cardio-protective benefits, aiding in the reduction of cardiotoxicity risks associated with cancer treatments [[37], [38], [39]]. Additionally, statins are recognized for their anti-inflammatory properties, which have been shown to confer protective effects against cardiovascular complications in breast cancer patients, further underscoring the importance of incorporating these agents into comprehensive cardio-oncology care [37,40].

Implementing strategies to minimize cardiotoxicity is crucial, not only for mitigating immediate health risks but also for preserving long-term quality of life. These strategies should be an integral part of the therapeutic regimen from the initial stages of treatment, ensuring that both oncological efficacy and cardiovascular health are maintained. By adopting such proactive measures, healthcare providers can better manage the dual challenges of cancer and cardiovascular disease, potentially transforming the survivorship experience for those undergoing breast cancer therapy.

#### 4.4.3 Policy interventions to reduce health disparities

Addressing the observed disparities necessitates robust policy interventions to ensure equitable access to cancer care and cardiovascular health services. Policies should focus on enhancing healthcare infrastructure, improving patient education, and ensuring that the benefits of medical advancements are equitably distributed, particularly in underserved and high-disparity regions.

### 4.5 Limitations

This study has several important limitations. First, the analysis relies on death certificate data from CDC WONDER, which is subject to cause-of-death misclassification, particularly in out-of-hospital settings. Some home deaths may represent home hospice cases rather than acute HF events, potentially including deaths from withdrawal of care or medical futility. Second, the dataset lacks granular clinical information, including cancer stage, treatment exposure, timing of diagnosis relative to death, and HF subtype. Consequently, causal relationships between breast cancer therapies and HF-related mortality cannot be established; findings represent descriptive associations only. Third, general population denominators, rather than breast cancer survivor-specific counts, are used, which may introduce estimation bias across racial and geographic subgroups with differing baseline demographics and cardiovascular risk profiles. Fourth, the predominance of deaths in patients ≥85 years limits the ability to disentangle age-related mortality from disease-specific effects, while younger age groups (25–64 years) were largely excluded from trend analyses due to CDC data suppression, restricting insights into early-onset cardiotoxicity patterns. Fifth, race and ethnicity analyses were limited by data suppression for Hispanic, NH Asian or Pacific Islander, and NH American Indian or Alaska Native populations. State-level and rural-urban trend analyses were restricted to 1999–2020 due to suppression of later data. Finally, CDC WONDER captures only mortality data; HF hospitalizations, emergency department visits, and non-fatal cardiotoxicity events are not represented, likely underestimating the true burden of HF morbidity in breast cancer survivors.

## 5 Conclusion

This study delineates the urgent need for integrated care strategies that encompass not only the oncological aspects of breast cancer but also the cardiovascular health of survivors. The recent increase, following prior decline, in HF-related mortality, with persistently higher rates among NH Black women and in specific geographic locales, amplifies the call for enhanced collaborative efforts across oncology, cardiology, public health, and policy domains to lessen disparities and improve the overall health outcomes of breast cancer survivors. These findings should be interpreted as descriptive associations given the limitations of the dataset. Future research using granular clinical data is needed to better understand underlying mechanisms and address disparities.

**Figure:**
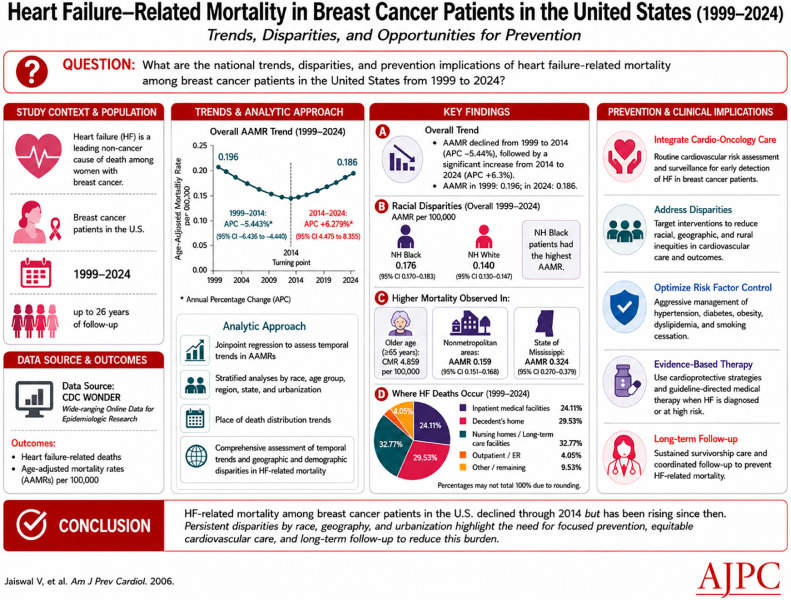
Central Illustration Heart Failure–Related Mortality in Breast Cancer Patients in the United States (1999–2024).

## Source of funding for this paper

None disclosed by the authors.

## Disclosure statement

Dr. Ana Barac reports serving on DSMB for ACI Clinical unrelated to this paper.

Dr. Fonarow reports consulting for Abbott, Amgen, AstraZeneca, Bayer, Boehinger Ingelheim,

Cytokinetics, Eli Lilly, Johnson & Johnson, Medtronic, Merck, Novartis, and Pfizer.

*Dr Banach* has received research grant(s)/support from Amgen, Daiichi Sankyo, Mylan/Viatris, and Sanofi, and has served as a speaker and consultant for Adamed, Amgen, Daiichi Sankyo, Esperion, Exceed Pharma, Kogen, KRKA, Menarini, Mylan, Novartis, Novo Nordisk, Pfizer, Polpharma, Sanofi-Aventis, Servier, Teva, Zentiva.

Rest of the authors have nothing to disclose.

## Data availability statement

The data underlying this article is available in the article and its online supplementary material.

## CRediT authorship contribution statement

**Vikash Jaiswal:** Writing – review & editing, Writing – original draft, Visualization, Validation, Investigation, Conceptualization. **Danisha Kumar:** Writing – review & editing, Writing – original draft, Methodology, Investigation, Formal analysis, Data curation. **Kriti Kalra:** Writing – review & editing, Writing – original draft. **Fakhar Latif:** Writing – original draft. **Yusra Minahil Nasir:** Writing – review & editing, Data curation. **Anupam Halder:** Writing – review & editing, Methodology. **Maciej Banach:** Writing – review & editing, Writing – original draft, Visualization, Validation, Supervision. **Garima Sharma:** Writing – review & editing, Writing – original draft, Visualization, Validation, Supervision. **Gregg C. Fonarow:** Writing – review & editing, Writing – original draft, Visualization, Validation, Supervision. **Ana Barac:** Writing – review & editing, Writing – original draft, Visualization, Validation, Supervision.

## Declaration of competing interest

The authors declare that they have no known competing financial interests or personal relationships that could have appeared to influence the work reported in this paper.

## Appendix Supplementary materials

Image, application 1
