# Supplementary material for: Trends in heart failure-related mortality among breast cancer patients in the United States from 1999 to 2024
Source: Am J Prev Cardiol. 2026 May 25;28:101680. doi: 10.1016/j.ajpc.2026.101680 (PMC13326086; doi:10.1016/j.ajpc.2026.101680)
Supplement: Supplementary file 1 [file mmc1.docx]

**Supplementary Legends**

**Figure S1**: JoinPoint Graph: Overall Heart Failure-related AAMRs per 100,000 in Breast Cancer Patients in the United States, 1999 to 2024. AAMRs= Age-Adjusted Mortality Rates; APC= Annual Percent Change.

**Figure S2:** Joinpoint Graph: Trends in Heart Failure related AAMR among Breast Cancer patients stratified by race in the United States, 1999-2024.

**Figure S3:** JoinPoint Graph: Trends in Heart Failure related AAMR among Breast Cancer patients stratified by census region in the United States, 1999-2024.

**Figure S4:** JoinPoint Graph: Trends in Heart Failure related AAMR among Breast Cancer patients stratified by urbanization in the United States, 1999-2020.

**Table S1:** Overall trends of Heart Failure Mortality among patients with Breast Cancer, 1999-2024.

**Table S2:** Race Stratified Heart Failure Related Mortality among Breast Cancer patients in the United States, 1999-2024.

**Table S3:** State Stratified Heart Failure Related Mortality among Breast Cancer Patients in the United States, 1999-2020.

**Table S4:** Census Region Stratified Heart Failure Related Mortality among Breast Cancer Patients in the United States, 1999-2024.

**Table S5:** Urbanization Stratified Heart Failure Related Mortality among Breast Cancer Patients in the United States, 1999-2020.

**Table S6:** Age Group Stratified Heart Failure Related Mortality among Breast Cancer Patients in the United States, 1999-2024.

**Table S7:** Total deaths due to Heart Failure in Breast Cancer Patients stratified by Place of Death in the United States, 1999-2024.


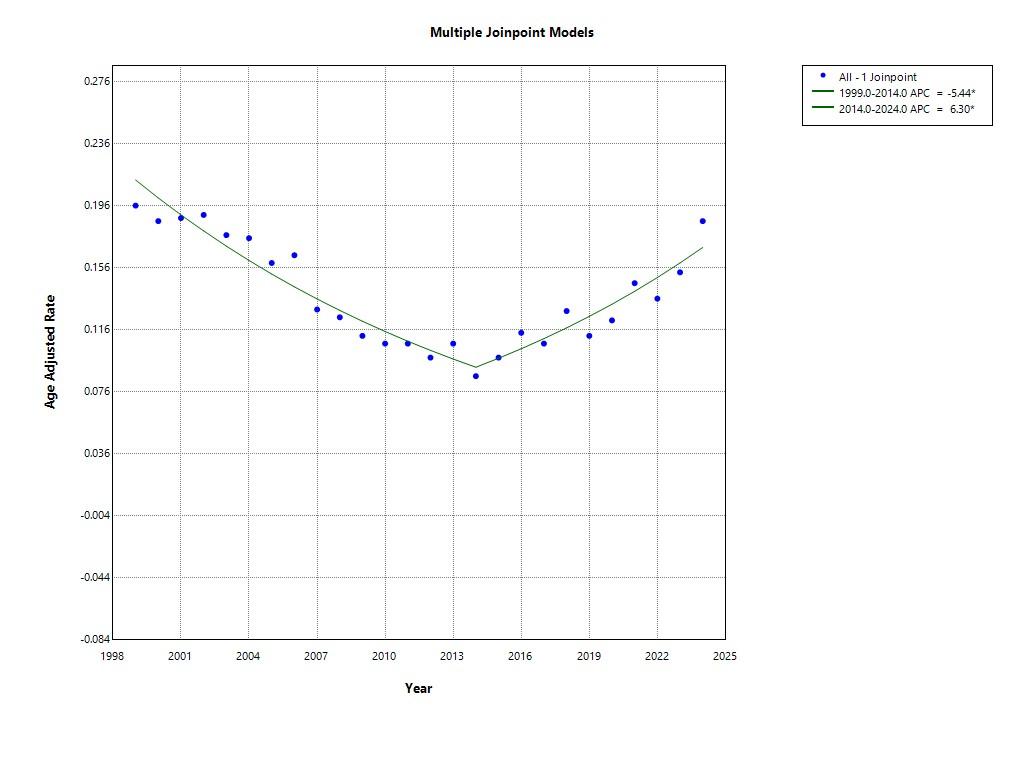


**Figure S1**: JoinPoint Graph: Overall Heart Failure-related AAMRs per 100,000 in Breast Cancer Patients in the United States, 1999 to 2024. AAMRs= Age-Adjusted Mortality Rates; APC= Annual Percent Change

* Indicates that Annual Percentage Change (APC) is significantly different from zero at α = 0.05.


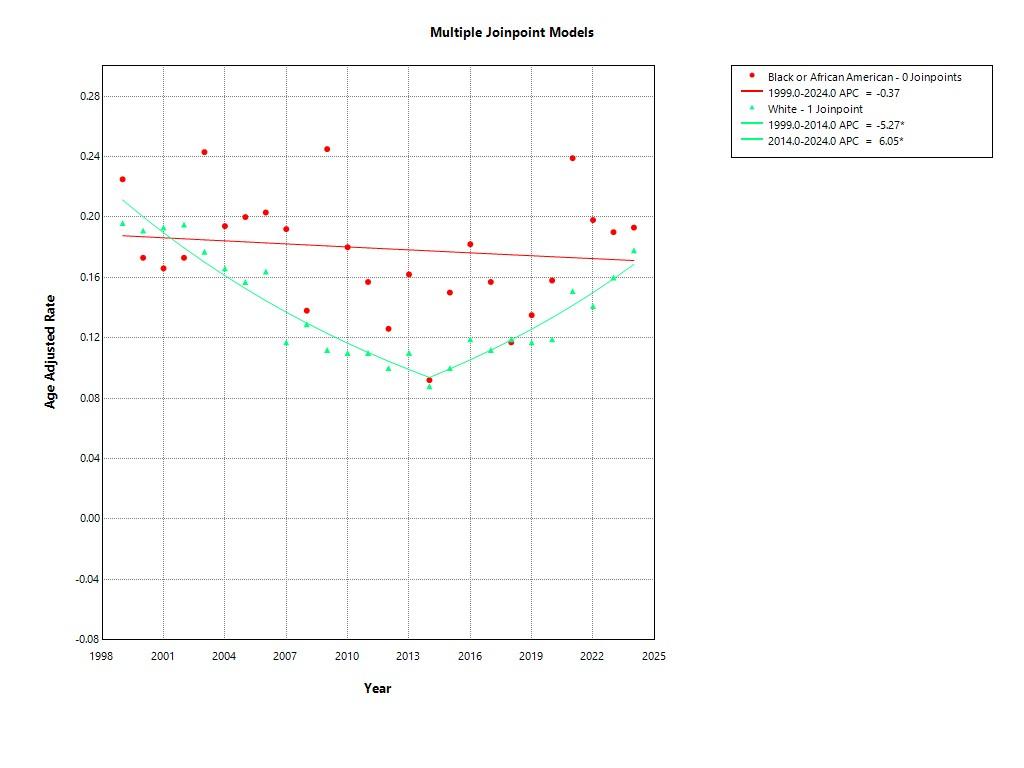


**Figure S2: Joinpoint Graph**: Trends in Heart Failure related AAMR among Breast Cancer patients stratified by race in the United States, 1999-2024. AAMR: Age-Adjusted Mortality Rate; APC: Annual Percent Change

* Indicates that Annual Percentage Change (APC) is significantly different from zero at α = 0.05.


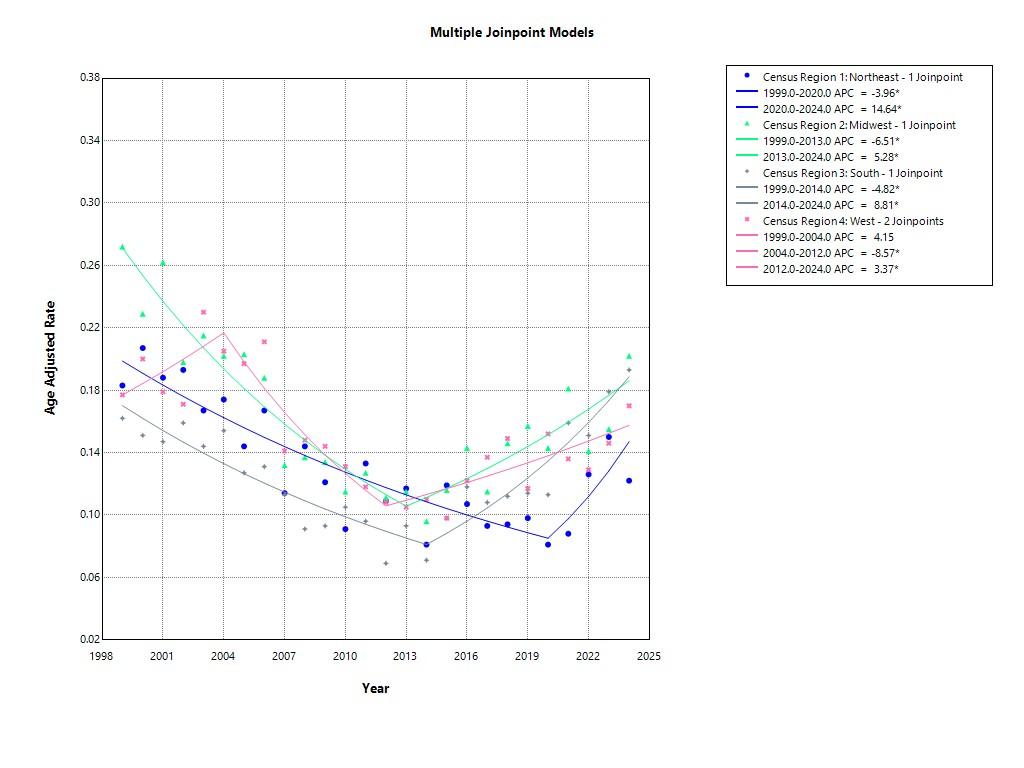


**Figure S3: JoinPoint Graph:** Trends in Heart Failure related AAMR among Breast Cancer patients stratified by census region in the United States, 1999-2024. AAMR: Age-Adjusted Mortality Rate; APC: Annual Percent Change

* Indicates that Annual Percentage Change (APC) is significantly different from zero at α = 0.05.


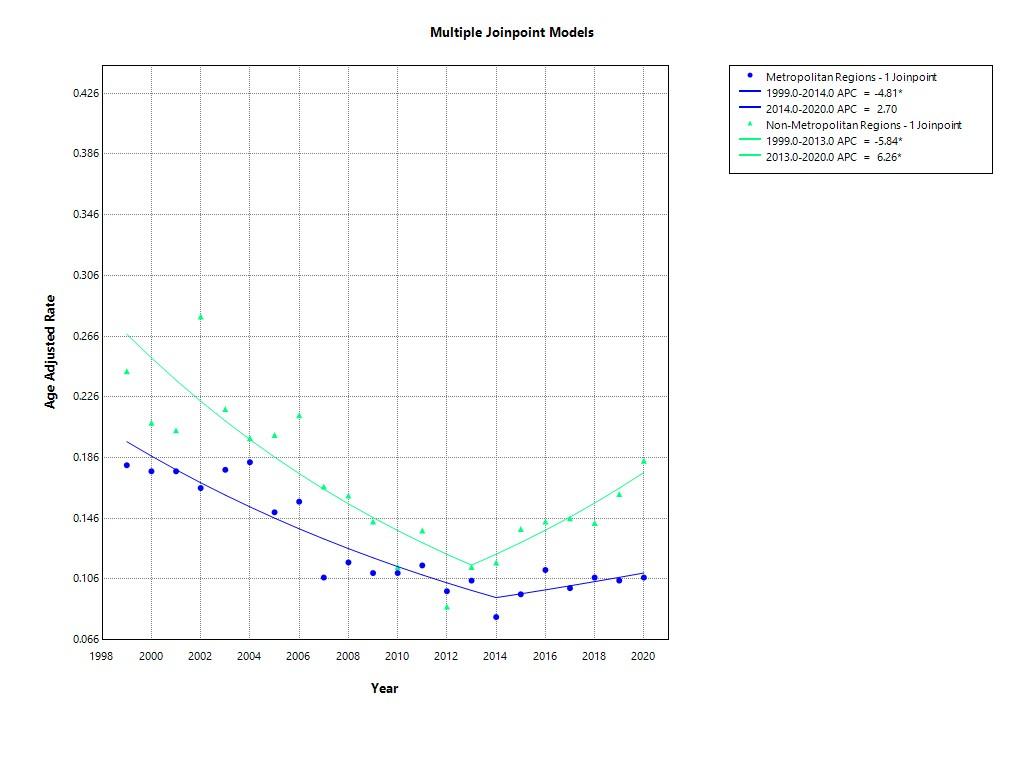


**Figure S4: JoinPoint Graph:** Trends in Heart Failure related AAMR among Breast Cancer patients stratified by urbanization in the United States, 1999-2020.** AAMR: Age-Adjusted Mortality Rate; APC: Annual Percent Change

* Indicates that Annual Percentage Change (APC) is significantly different from zero at α = 0.05.

**Detailed reports on mortality data of the metropolitan and nonmetropolitan regions, including AAMRs and population, are suppressed or unreliable in the CDC database for the years 2021-2024, and therefore have been excluded from this graph.

| **Year** | **Deaths** | **Population** | **Age Adjusted Mortality Rate** | **Age Adjusted Mortality Rate Lower 95% Confidence Interval** | **Age Adjusted Mortality Rate Upper 95% Confidence Interval** |
| --- | --- | --- | --- | --- | --- |
| 1999 | 353 | 180408769 | 0.196 | 0.175 | 0.216 |
| 2000 | 344 | 181984640 | 0.186 | 0.166 | 0.206 |
| 2001 | 347 | 184305128 | 0.188 | 0.168 | 0.209 |
| 2002 | 345 | 186208028 | 0.19 | 0.17 | 0.21 |
| 2003 | 353 | 188090429 | 0.177 | 0.158 | 0.196 |
| 2004 | 339 | 190205384 | 0.175 | 0.156 | 0.194 |
| 2005 | 321 | 192551384 | 0.159 | 0.142 | 0.177 |
| 2006 | 346 | 195019359 | 0.164 | 0.147 | 0.182 |
| 2007 | 265 | 197403777 | 0.129 | 0.113 | 0.146 |
| 2008 | 275 | 199795090 | 0.124 | 0.109 | 0.139 |
| 2009 | 272 | 202107016 | 0.112 | 0.098 | 0.126 |
| 2010 | 251 | 203891983 | 0.107 | 0.094 | 0.121 |
| 2011 | 269 | 206592936 | 0.107 | 0.094 | 0.121 |
| 2012 | 234 | 208826037 | 0.098 | 0.085 | 0.111 |
| 2013 | 272 | 211085314 | 0.107 | 0.094 | 0.121 |
| 2014 | 214 | 213809280 | 0.086 | 0.074 | 0.098 |
| 2015 | 278 | 216553817 | 0.098 | 0.086 | 0.11 |
| 2016 | 308 | 218641417 | 0.114 | 0.101 | 0.128 |
| 2017 | 300 | 221447331 | 0.107 | 0.095 | 0.12 |
| 2018 | 324 | 223311190 | 0.128 | 0.114 | 0.142 |
| 2019 | 334 | 224981167 | 0.112 | 0.1 | 0.125 |
| 2020 | 345 | 226635013 | 0.122 | 0.109 | 0.136 |
| 2021 | 386 | 228238412 | 0.146 | 0.131 | 0.162 |
| 2022 | 385 | 229508599 | 0.136 | 0.122 | 0.151 |
| 2023 | 444 | 231529762 | 0.153 | 0.138 | 0.17 |
| 2024 | 494 | 235615087 | 0.186 | 0.17 | 0.202 |

**Table S1:** Overall trends of Heart Failure Mortality among patients with Breast Cancer, 1999-2024

| **Race** | **Year** | **Deaths** | **Population** | **Age Adjusted Mortality Rate** | **Age Adjusted Mortality Rate Lower 95% Confidence Interval** | **Age Adjusted Mortality Rate Upper 95% Confidence Interval** |
| --- | --- | --- | --- | --- | --- | --- |
| Black or African American | 1999 | 34 | 20503656 | 0.225 | 0.153 | 0.319 |
| Black or African American | 2000 | 26 | 20775703 | 0.173 | 0.112 | 0.255 |
| Black or African American | 2001 | 25 | 21181572 | 0.166 | 0.105 | 0.249 |
| Black or African American | 2002 | 28 | 21522362 | 0.173 | 0.113 | 0.254 |
| Black or African American | 2003 | 37 | 21858880 | 0.243 | 0.169 | 0.338 |
| Black or African American | 2004 | 34 | 22258267 | 0.194 | 0.131 | 0.277 |
| Black or African American | 2005 | 33 | 22690893 | 0.2 | 0.135 | 0.285 |
| Black or African American | 2006 | 33 | 23137867 | 0.203 | 0.137 | 0.289 |
| Black or African American | 2007 | 35 | 23577304 | 0.192 | 0.132 | 0.272 |
| Black or African American | 2008 | 24 | 24018935 | 0.138 | 0.087 | 0.207 |
| Black or African American | 2009 | 43 | 24456946 | 0.245 | 0.176 | 0.332 |
| Black or African American | 2010 | 32 | 24802842 | 0.18 | 0.12 | 0.258 |
| Black or African American | 2011 | 33 | 25288869 | 0.157 | 0.106 | 0.224 |
| Black or African American | 2012 | 27 | 25724605 | 0.126 | 0.081 | 0.187 |
| Black or African American | 2013 | 32 | 26175234 | 0.162 | 0.109 | 0.232 |
| Black or African American | 2014 | 19 | 26742836 | 0.092 | 0.049 | 0.136 |
| Black or African American | 2015 | 37 | 27316901 | 0.15 | 0.104 | 0.21 |
| Black or African American | 2016 | 43 | 27833992 | 0.182 | 0.13 | 0.249 |
| Black or African American | 2017 | 36 | 28455786 | 0.157 | 0.108 | 0.221 |
| Black or African American | 2018 | 30 | 28919914 | 0.117 | 0.078 | 0.169 |
| Black or African American | 2019 | 36 | 29385485 | 0.135 | 0.093 | 0.19 |
| Black or African American | 2020 | 42 | 29818678 | 0.158 | 0.113 | 0.216 |
| Black or African American | 2021 | 55 | 29240362 | 0.239 | 0.182 | 0.309 |
| Black or African American | 2022 | 49 | 29454399 | 0.198 | 0.148 | 0.26 |
| Black or African American | 2023 | 55 | 29874145 | 0.19 | 0.139 | 0.254 |
| Black or African American | 2024 | 53 | 30536299 | 0.193 | 0.144 | 0.254 |
| White | 1999 | 318 | 151306036 | 0.196 | 0.174 | 0.218 |
| White | 2000 | 314 | 152222052 | 0.191 | 0.169 | 0.213 |
| White | 2001 | 319 | 153608264 | 0.193 | 0.172 | 0.215 |
| White | 2002 | 315 | 154739951 | 0.195 | 0.173 | 0.217 |
| White | 2003 | 309 | 155864104 | 0.177 | 0.156 | 0.197 |
| White | 2004 | 301 | 157144830 | 0.166 | 0.147 | 0.186 |
| White | 2005 | 285 | 158595744 | 0.157 | 0.138 | 0.176 |
| White | 2006 | 311 | 160142968 | 0.164 | 0.146 | 0.183 |
| White | 2007 | 228 | 161626163 | 0.117 | 0.102 | 0.133 |
| White | 2008 | 247 | 163116422 | 0.129 | 0.113 | 0.145 |
| White | 2009 | 224 | 164541687 | 0.112 | 0.097 | 0.127 |
| White | 2010 | 215 | 165644483 | 0.11 | 0.095 | 0.125 |
| White | 2011 | 235 | 167382563 | 0.11 | 0.096 | 0.124 |
| White | 2012 | 202 | 168663175 | 0.1 | 0.086 | 0.115 |
| White | 2013 | 235 | 169982244 | 0.11 | 0.095 | 0.125 |
| White | 2014 | 191 | 171489057 | 0.088 | 0.075 | 0.101 |
| White | 2015 | 235 | 173031552 | 0.1 | 0.087 | 0.114 |
| White | 2016 | 257 | 174238249 | 0.119 | 0.104 | 0.134 |
| White | 2017 | 260 | 175747095 | 0.112 | 0.098 | 0.126 |
| White | 2018 | 291 | 176757620 | 0.119 | 0.105 | 0.134 |
| White | 2019 | 290 | 177619677 | 0.117 | 0.103 | 0.131 |
| White | 2020 | 298 | 178513471 | 0.119 | 0.105 | 0.133 |
| White | 2021 | 319 | 177226927 | 0.151 | 0.135 | 0.168 |
| White | 2022 | 329 | 177627950 | 0.141 | 0.125 | 0.158 |
| White | 2023 | 380 | 178598830 | 0.16 | 0.144 | 0.179 |
| White | 2024 | 420 | 180588822 | 0.178 | 0.161 | 0.197 |

**Table S2:** Race Stratified Heart Failure Related Mortality among Breast Cancer patients in the United States, 1999-2024.*

*Detailed reports on race populations such as Hispanic or Latino, Asian or Pacific Islander, American Indian or Alaska Native are suppressed or unreliable in the CDC database and therefore have been excluded from this table.

| **State** | **Deaths** | **Population** | **Age Adjusted Mortality Rate** |
| --- | --- | --- | --- |
| Alabama | 89 | 68915349 | 0.126 |
| Alaska | Suppressed | 9623822 | Suppressed |
| Arizona | 73 | 90089006 | 0.069 |
| Arkansas | 56 | 41711420 | 0.121 |
| California | 831 | 530625638 | 0.16 |
| Colorado | 139 | 73189174 | 0.216 |
| Connecticut | 79 | 53012824 | 0.112 |
| Delaware | 17 | 13210778 | Unreliable |
| District of Columbia | 15 | 9448827 | Unreliable |
| Florida | 204 | 289701852 | 0.053 |
| Georgia | 150 | 135415629 | 0.133 |
| Hawaii | 30 | 20066183 | 0.126 |
| Idaho | 35 | 21392071 | 0.155 |
| Illinois | 257 | 184489320 | 0.119 |
| Indiana | 152 | 92386904 | 0.139 |
| Iowa | 87 | 44030204 | 0.136 |
| Kansas | 73 | 39931570 | 0.147 |
| Kentucky | 80 | 63061451 | 0.114 |
| Louisiana | 79 | 64895449 | 0.11 |
| Maine | 29 | 20521512 | 0.106 |
| Maryland | 101 | 84902583 | 0.117 |
| Massachusetts | 183 | 99096796 | 0.145 |
| Michigan | 238 | 145550610 | 0.136 |
| Minnesota | 161 | 77002440 | 0.177 |
| Mississippi | 141 | 41496865 | 0.324 |
| Missouri | 148 | 86518061 | 0.138 |
| Montana | 29 | 14577633 | 0.171 |
| Nebraska | 90 | 25869503 | 0.261 |
| Nevada | 19 | 38580189 | Unreliable |
| New Hampshire | 32 | 19856602 | 0.148 |
| New Jersey | 188 | 130947870 | 0.117 |
| New Mexico | 30 | 28568524 | 0.098 |
| New York | 448 | 288624005 | 0.134 |
| North Carolina | 173 | 137233223 | 0.124 |
| North Dakota | 18 | 9822062 | Unreliable |
| Ohio | 365 | 169651360 | 0.174 |
| Oklahoma | 150 | 53110132 | 0.254 |
| Oregon | 127 | 57214641 | 0.194 |
| Pennsylvania | 329 | 189975429 | 0.126 |
| Rhode Island | 22 | 15765032 | 0.103 |
| South Carolina | 89 | 67227226 | 0.125 |
| South Dakota | 27 | 11603220 | 0.172 |
| Tennessee | 105 | 92689763 | 0.1 |
| Texas | 408 | 344632461 | 0.132 |
| Utah | 43 | 34057911 | 0.153 |
| Vermont | 15 | 9393709 | Unreliable |
| Virginia | 144 | 116579933 | 0.119 |
| Washington | 141 | 98931323 | 0.14 |
| West Virginia | 49 | 28023276 | 0.159 |
| Wisconsin | 183 | 82712057 | 0.193 |
| Wyoming | 10 | 7921067 | Unreliable |

**Table S3:** State Stratified Heart Failure Related Mortality among Breast Cancer Patients in the United States, 1999-2020.*

*Detailed reports on mortality data of the states, including AAMRs and population, are suppressed or unreliable in the CDC database for the years 2021-2024, and therefore have been excluded from this table.

| **Census Region** | **Year** | **Deaths** | **Population** | **Age Adjusted Mortality Rate** | **Age Adjusted Mortality Rate Lower 95% Confidence Interval** | **Age Adjusted Mortality Rate Upper 95% Confidence Interval** |
| --- | --- | --- | --- | --- | --- | --- |
| Northeast | 1999 | 72 | 35633134 | 0.183 | 0.143 | 0.23 |
| Northeast | 2000 | 81 | 35788687 | 0.207 | 0.164 | 0.258 |
| Northeast | 2001 | 76 | 36006250 | 0.188 | 0.148 | 0.236 |
| Northeast | 2002 | 81 | 36185082 | 0.193 | 0.153 | 0.241 |
| Northeast | 2003 | 68 | 36346948 | 0.167 | 0.129 | 0.212 |
| Northeast | 2004 | 69 | 36462699 | 0.174 | 0.135 | 0.22 |
| Northeast | 2005 | 59 | 36559788 | 0.144 | 0.109 | 0.188 |
| Northeast | 2006 | 72 | 36682176 | 0.167 | 0.13 | 0.211 |
| Northeast | 2007 | 48 | 36846338 | 0.114 | 0.084 | 0.152 |
| Northeast | 2008 | 66 | 37084149 | 0.144 | 0.11 | 0.185 |
| Northeast | 2009 | 57 | 37339597 | 0.121 | 0.091 | 0.159 |
| Northeast | 2010 | 43 | 37543347 | 0.091 | 0.064 | 0.125 |
| Northeast | 2011 | 61 | 37864117 | 0.133 | 0.101 | 0.173 |
| Northeast | 2012 | 56 | 38158527 | 0.109 | 0.081 | 0.143 |
| Northeast | 2013 | 61 | 38437194 | 0.117 | 0.089 | 0.152 |
| Northeast | 2014 | 43 | 38710627 | 0.081 | 0.059 | 0.11 |
| Northeast | 2015 | 58 | 38965872 | 0.119 | 0.09 | 0.155 |
| Northeast | 2016 | 55 | 39040202 | 0.107 | 0.081 | 0.14 |
| Northeast | 2017 | 49 | 39417175 | 0.093 | 0.068 | 0.125 |
| Northeast | 2018 | 51 | 39321978 | 0.094 | 0.069 | 0.124 |
| Northeast | 2019 | 53 | 39381333 | 0.098 | 0.073 | 0.129 |
| Northeast | 2020 | 46 | 39418559 | 0.081 | 0.059 | 0.109 |
| Northeast | 2021 | 49 | 40339548 | 0.088 | 0.062 | 0.122 |
| Northeast | 2022 | 62 | 40289184 | 0.126 | 0.098 | 0.161 |
| Northeast | 2023 | 78 | 40402747 | 0.15 | 0.118 | 0.19 |
| Northeast | 2024 | 68 | 41034160 | 0.122 | 0.093 | 0.158 |
| Midwest | 1999 | 115 | 41293967 | 0.272 | 0.221 | 0.322 |
| Midwest | 2000 | 95 | 41504992 | 0.229 | 0.185 | 0.28 |
| Midwest | 2001 | 115 | 41762990 | 0.262 | 0.213 | 0.31 |
| Midwest | 2002 | 95 | 41970731 | 0.198 | 0.159 | 0.243 |
| Midwest | 2003 | 99 | 42200881 | 0.215 | 0.174 | 0.263 |
| Midwest | 2004 | 89 | 42455980 | 0.202 | 0.162 | 0.25 |
| Midwest | 2005 | 95 | 42748579 | 0.203 | 0.164 | 0.25 |
| Midwest | 2006 | 96 | 43084311 | 0.188 | 0.151 | 0.231 |
| Midwest | 2007 | 68 | 43424366 | 0.132 | 0.102 | 0.168 |
| Midwest | 2008 | 71 | 43718509 | 0.137 | 0.106 | 0.173 |
| Midwest | 2009 | 68 | 44010460 | 0.134 | 0.104 | 0.171 |
| Midwest | 2010 | 61 | 44248465 | 0.115 | 0.088 | 0.148 |
| Midwest | 2011 | 67 | 44584105 | 0.127 | 0.098 | 0.162 |
| Midwest | 2012 | 63 | 44817227 | 0.111 | 0.085 | 0.144 |
| Midwest | 2013 | 63 | 45090597 | 0.115 | 0.088 | 0.148 |
| Midwest | 2014 | 53 | 45360409 | 0.096 | 0.071 | 0.126 |
| Midwest | 2015 | 69 | 45628315 | 0.116 | 0.09 | 0.148 |
| Midwest | 2016 | 78 | 45802491 | 0.143 | 0.112 | 0.179 |
| Midwest | 2017 | 68 | 46143783 | 0.115 | 0.088 | 0.146 |
| Midwest | 2018 | 85 | 46405110 | 0.146 | 0.115 | 0.182 |
| Midwest | 2019 | 98 | 46589364 | 0.157 | 0.127 | 0.193 |
| Midwest | 2020 | 88 | 46721679 | 0.143 | 0.114 | 0.177 |
| Midwest | 2021 | 102 | 47032092 | 0.181 | 0.147 | 0.223 |
| Midwest | 2022 | 83 | 47087294 | 0.141 | 0.111 | 0.177 |
| Midwest | 2023 | 90 | 47312396 | 0.155 | 0.124 | 0.193 |
| Midwest | 2024 | 117 | 47893529 | 0.202 | 0.167 | 0.243 |
| South | 1999 | 106 | 64108630 | 0.162 | 0.13 | 0.194 |
| South | 2000 | 98 | 64843390 | 0.151 | 0.121 | 0.185 |
| South | 2001 | 94 | 65874012 | 0.147 | 0.118 | 0.18 |
| South | 2002 | 107 | 66758178 | 0.159 | 0.128 | 0.19 |
| South | 2003 | 101 | 67639133 | 0.144 | 0.114 | 0.173 |
| South | 2004 | 101 | 68718205 | 0.154 | 0.123 | 0.184 |
| South | 2005 | 90 | 69951038 | 0.127 | 0.101 | 0.158 |
| South | 2006 | 93 | 71198336 | 0.131 | 0.105 | 0.162 |
| South | 2007 | 82 | 72387467 | 0.113 | 0.089 | 0.142 |
| South | 2008 | 72 | 73529724 | 0.091 | 0.07 | 0.116 |
| South | 2009 | 78 | 74596130 | 0.093 | 0.072 | 0.118 |
| South | 2010 | 86 | 75419767 | 0.105 | 0.083 | 0.131 |
| South | 2011 | 85 | 76650163 | 0.096 | 0.076 | 0.12 |
| South | 2012 | 61 | 77681893 | 0.069 | 0.053 | 0.09 |
| South | 2013 | 89 | 78693557 | 0.093 | 0.073 | 0.117 |
| South | 2014 | 61 | 79945474 | 0.071 | 0.054 | 0.091 |
| South | 2015 | 95 | 81260812 | 0.098 | 0.078 | 0.121 |
| South | 2016 | 106 | 82405493 | 0.118 | 0.095 | 0.141 |
| South | 2017 | 104 | 83659214 | 0.108 | 0.086 | 0.13 |
| South | 2018 | 108 | 84717024 | 0.112 | 0.091 | 0.134 |
| South | 2019 | 109 | 85606773 | 0.114 | 0.092 | 0.136 |
| South | 2020 | 124 | 86611804 | 0.113 | 0.092 | 0.133 |
| South | 2021 | 158 | 86926028 | 0.159 | 0.134 | 0.188 |
| South | 2022 | 157 | 87978320 | 0.151 | 0.127 | 0.178 |
| South | 2023 | 186 | 89219544 | 0.179 | 0.154 | 0.208 |
| South | 2024 | 198 | 91136871 | 0.193 | 0.167 | 0.222 |
| West | 1999 | 60 | 39373038 | 0.177 | 0.135 | 0.228 |
| West | 2000 | 70 | 39847571 | 0.2 | 0.156 | 0.253 |
| West | 2001 | 62 | 40661876 | 0.179 | 0.137 | 0.23 |
| West | 2002 | 62 | 41294037 | 0.171 | 0.131 | 0.219 |
| West | 2003 | 85 | 41903467 | 0.23 | 0.183 | 0.285 |
| West | 2004 | 80 | 42568500 | 0.205 | 0.161 | 0.256 |
| West | 2005 | 77 | 43291979 | 0.197 | 0.156 | 0.247 |
| West | 2006 | 85 | 44054536 | 0.211 | 0.169 | 0.262 |
| West | 2007 | 67 | 44745606 | 0.141 | 0.108 | 0.181 |
| West | 2008 | 66 | 45462708 | 0.148 | 0.114 | 0.189 |
| West | 2009 | 69 | 46160829 | 0.144 | 0.111 | 0.183 |
| West | 2010 | 61 | 46680404 | 0.131 | 0.1 | 0.17 |
| West | 2011 | 56 | 47494551 | 0.118 | 0.088 | 0.154 |
| West | 2012 | 54 | 48168390 | 0.108 | 0.081 | 0.142 |
| West | 2013 | 59 | 48863966 | 0.105 | 0.079 | 0.137 |
| West | 2014 | 57 | 49792770 | 0.11 | 0.083 | 0.143 |
| West | 2015 | 56 | 50698818 | 0.098 | 0.073 | 0.128 |
| West | 2016 | 69 | 51393231 | 0.122 | 0.094 | 0.154 |
| West | 2017 | 79 | 52227159 | 0.137 | 0.107 | 0.172 |
| West | 2018 | 80 | 52867078 | 0.149 | 0.118 | 0.186 |
| West | 2019 | 74 | 53403697 | 0.117 | 0.091 | 0.148 |
| West | 2020 | 87 | 53882971 | 0.152 | 0.121 | 0.188 |
| West | 2021 | 77 | 53940744 | 0.136 | 0.107 | 0.172 |
| West | 2022 | 83 | 54153801 | 0.129 | 0.101 | 0.163 |
| West | 2023 | 90 | 54595075 | 0.146 | 0.117 | 0.182 |
| West | 2024 | 111 | 55550527 | 0.17 | 0.138 | 0.207 |

**Table S4:** Census Region Stratified Heart Failure Related Mortality among Breast Cancer Patients in the United States, 1999-2024

| **Urban-Rural Classification** | **Year** | **Deaths** | **Population** | **Age Adjusted Mortality Rate** | **Age Adjusted Mortality Rate Lower 95% Confidence Interval** | **Age Adjusted Mortality Rate Upper 95% Confidence Interval** |
| --- | --- | --- | --- | --- | --- | --- |
| Metropolitan Regions | 1999 | 268 | 151245342 | 0.181 | 0.159 | 0.204 |
| Metropolitan Regions | 2000 | 271 | 152658699 | 0.177 | 0.155 | 0.198 |
| Metropolitan Regions | 2001 | 274 | 154896258 | 0.177 | 0.155 | 0.198 |
| Metropolitan Regions | 2002 | 244 | 156659051 | 0.166 | 0.144 | 0.187 |
| Metropolitan Regions | 2003 | 272 | 158367715 | 0.178 | 0.157 | 0.2 |
| Metropolitan Regions | 2004 | 268 | 160272097 | 0.183 | 0.161 | 0.205 |
| Metropolitan Regions | 2005 | 250 | 162371826 | 0.15 | 0.131 | 0.169 |
| Metropolitan Regions | 2006 | 264 | 164523389 | 0.157 | 0.138 | 0.177 |
| Metropolitan Regions | 2007 | 204 | 166650886 | 0.107 | 0.092 | 0.123 |
| Metropolitan Regions | 2008 | 210 | 168826027 | 0.117 | 0.1 | 0.133 |
| Metropolitan Regions | 2009 | 216 | 170965574 | 0.11 | 0.095 | 0.125 |
| Metropolitan Regions | 2010 | 204 | 172591105 | 0.11 | 0.094 | 0.125 |
| Metropolitan Regions | 2011 | 211 | 175204532 | 0.115 | 0.099 | 0.131 |
| Metropolitan Regions | 2012 | 199 | 177423676 | 0.098 | 0.084 | 0.112 |
| Metropolitan Regions | 2013 | 221 | 179634449 | 0.105 | 0.091 | 0.12 |
| Metropolitan Regions | 2014 | 166 | 182304016 | 0.081 | 0.068 | 0.094 |
| Metropolitan Regions | 2015 | 218 | 184959306 | 0.096 | 0.082 | 0.109 |
| Metropolitan Regions | 2016 | 247 | 186963190 | 0.112 | 0.097 | 0.127 |
| Metropolitan Regions | 2017 | 231 | 189675062 | 0.1 | 0.087 | 0.114 |
| Metropolitan Regions | 2018 | 265 | 191415024 | 0.107 | 0.094 | 0.121 |
| Metropolitan Regions | 2019 | 259 | 193006488 | 0.105 | 0.092 | 0.118 |
| Metropolitan Regions | 2020 | 264 | 194600110 | 0.107 | 0.094 | 0.121 |
| Nonmetropolitan Regions | 1999 | 85 | 29163427 | 0.243 | 0.194 | 0.301 |
| Nonmetropolitan Regions | 2000 | 73 | 29325941 | 0.209 | 0.164 | 0.264 |
| Nonmetropolitan Regions | 2001 | 73 | 29408870 | 0.204 | 0.159 | 0.256 |
| Nonmetropolitan Regions | 2002 | 101 | 29548977 | 0.279 | 0.223 | 0.334 |
| Nonmetropolitan Regions | 2003 | 81 | 29722714 | 0.218 | 0.172 | 0.273 |
| Nonmetropolitan Regions | 2004 | 71 | 29933287 | 0.199 | 0.154 | 0.251 |
| Nonmetropolitan Regions | 2005 | 71 | 30179558 | 0.201 | 0.156 | 0.254 |
| Nonmetropolitan Regions | 2006 | 82 | 30495970 | 0.214 | 0.169 | 0.267 |
| Nonmetropolitan Regions | 2007 | 61 | 30752891 | 0.167 | 0.127 | 0.215 |
| Nonmetropolitan Regions | 2008 | 65 | 30969063 | 0.161 | 0.123 | 0.206 |
| Nonmetropolitan Regions | 2009 | 56 | 31141442 | 0.144 | 0.109 | 0.187 |
| Nonmetropolitan Regions | 2010 | 47 | 31300878 | 0.114 | 0.083 | 0.153 |
| Nonmetropolitan Regions | 2011 | 58 | 31388404 | 0.138 | 0.104 | 0.18 |
| Nonmetropolitan Regions | 2012 | 35 | 31402361 | 0.088 | 0.061 | 0.123 |
| Nonmetropolitan Regions | 2013 | 51 | 31450865 | 0.114 | 0.083 | 0.153 |
| Nonmetropolitan Regions | 2014 | 48 | 31505264 | 0.117 | 0.086 | 0.156 |
| Nonmetropolitan Regions | 2015 | 60 | 31594511 | 0.139 | 0.106 | 0.18 |
| Nonmetropolitan Regions | 2016 | 61 | 31678227 | 0.144 | 0.11 | 0.185 |
| Nonmetropolitan Regions | 2017 | 69 | 31772269 | 0.146 | 0.112 | 0.186 |
| Nonmetropolitan Regions | 2018 | 59 | 31896166 | 0.143 | 0.108 | 0.185 |
| Nonmetropolitan Regions | 2019 | 75 | 31974679 | 0.162 | 0.126 | 0.205 |
| Nonmetropolitan Regions | 2020 | 81 | 32028405 | 0.184 | 0.146 | 0.229 |

**Table S5:** Urbanization Stratified Heart Failure Related Mortality among Breast Cancer Patients in the United States, 1999-2020.*

*Detailed reports on mortality data of the metropolitan and nonmetropolitan regions, including AAMRs and population, are suppressed or unreliable in the CDC database for the years 2021-2024, and therefore have been excluded from this table.

| **Ten-Year Age Groups** | **Year** | **Deaths** | **Population** | **Crude Rate** | **Crude Rate Lower 95% Confidence Interval** | **Crude Rate Upper 95% Confidence Interval** |
| --- | --- | --- | --- | --- | --- | --- |
| 25-34 years | 1999 | Suppressed | 40178406 | Suppressed | Suppressed | Suppressed |
| 25-34 years | 2000 | 0 | 39891724 | Unreliable | 0 | 0 |
| 25-34 years | 2001 | 0 | 39471522 | Unreliable | 0 | 0 |
| 25-34 years | 2002 | 0 | 39349646 | Unreliable | 0 | 0 |
| 25-34 years | 2003 | 0 | 39243795 | Unreliable | 0 | 0 |
| 25-34 years | 2004 | 0 | 39266556 | Unreliable | 0 | 0 |
| 25-34 years | 2005 | 0 | 39258647 | Unreliable | 0 | 0 |
| 25-34 years | 2006 | 0 | 39395179 | Unreliable | 0 | 0 |
| 25-34 years | 2007 | 0 | 39713463 | Unreliable | 0 | 0 |
| 25-34 years | 2008 | 0 | 40207473 | Unreliable | 0 | 0 |
| 25-34 years | 2009 | 0 | 40723342 | Unreliable | 0 | 0 |
| 25-34 years | 2010 | 0 | 41063948 | Unreliable | 0 | 0 |
| 25-34 years | 2011 | 0 | 41790498 | Unreliable | 0 | 0 |
| 25-34 years | 2012 | 0 | 42309321 | Unreliable | 0 | 0 |
| 25-34 years | 2013 | Suppressed | 42844587 | Suppressed | Suppressed | Suppressed |
| 25-34 years | 2014 | 0 | 43516504 | Unreliable | 0 | 0 |
| 25-34 years | 2015 | Suppressed | 44137202 | Suppressed | Suppressed | Suppressed |
| 25-34 years | 2016 | 0 | 44677243 | Unreliable | 0 | 0 |
| 25-34 years | 2017 | 0 | 45342672 | Unreliable | 0 | 0 |
| 25-34 years | 2018 | 0 | 45697774 | Unreliable | 0 | 0 |
| 25-34 years | 2019 | 0 | 45940321 | Unreliable | 0 | 0 |
| 25-34 years | 2020 | 0 | 46069646 | Unreliable | 0 | 0 |
| 25-34 years | 2021 | 0 | 45495105 | 0 | 0 | 0 |
| 25-34 years | 2022 | 0 | 45501300 | 0 | 0 | 0 |
| 25-34 years | 2023 | 0 | 45542516 | 0 | 0 | 0 |
| 25-34 years | 2024 | 0 | 46453864 | 0 | 0 | 0 |
| 35-44 years | 1999 | 0 | 45076677 | Unreliable | 0 | 0 |
| 35-44 years | 2000 | Suppressed | 45148527 | Suppressed | Suppressed | Suppressed |
| 35-44 years | 2001 | 0 | 45051752 | Unreliable | 0 | 0 |
| 35-44 years | 2002 | 0 | 44640649 | Unreliable | 0 | 0 |
| 35-44 years | 2003 | Suppressed | 44154206 | Suppressed | Suppressed | Suppressed |
| 35-44 years | 2004 | 0 | 43800275 | Unreliable | 0 | 0 |
| 35-44 years | 2005 | Suppressed | 43505538 | Suppressed | Suppressed | Suppressed |
| 35-44 years | 2006 | Suppressed | 43243801 | Suppressed | Suppressed | Suppressed |
| 35-44 years | 2007 | Suppressed | 42796230 | Suppressed | Suppressed | Suppressed |
| 35-44 years | 2008 | 0 | 42192486 | Unreliable | 0 | 0 |
| 35-44 years | 2009 | 0 | 41487811 | Unreliable | 0 | 0 |
| 35-44 years | 2010 | Suppressed | 41070606 | Suppressed | Suppressed | Suppressed |
| 35-44 years | 2011 | 0 | 40627954 | Unreliable | 0 | 0 |
| 35-44 years | 2012 | Suppressed | 40516420 | Suppressed | Suppressed | Suppressed |
| 35-44 years | 2013 | Suppressed | 40452690 | Suppressed | Suppressed | Suppressed |
| 35-44 years | 2014 | Suppressed | 40513133 | Suppressed | Suppressed | Suppressed |
| 35-44 years | 2015 | 0 | 40589783 | Unreliable | 0 | 0 |
| 35-44 years | 2016 | Suppressed | 40470156 | Suppressed | Suppressed | Suppressed |
| 35-44 years | 2017 | Suppressed | 40875370 | Suppressed | Suppressed | Suppressed |
| 35-44 years | 2018 | Suppressed | 41277888 | Suppressed | Suppressed | Suppressed |
| 35-44 years | 2019 | Suppressed | 41659144 | Suppressed | Suppressed | Suppressed |
| 35-44 years | 2020 | Suppressed | 42136192 | Suppressed | Suppressed | Suppressed |
| 35-44 years | 2021 | Suppressed | 43403854 | Suppressed | Suppressed | Suppressed |
| 35-44 years | 2022 | 0 | 43695365 | 0 | 0 | 0 |
| 35-44 years | 2023 | Suppressed | 44390693 | Suppressed | Suppressed | Suppressed |
| 35-44 years | 2024 | Suppressed | 45539224 | Suppressed | Suppressed | Suppressed |
| 45-54 years | 1999 | Suppressed | 36577819 | Suppressed | Suppressed | Suppressed |
| 45-54 years | 2000 | Suppressed | 37677952 | Suppressed | Suppressed | Suppressed |
| 45-54 years | 2001 | Suppressed | 39386268 | Suppressed | Suppressed | Suppressed |
| 45-54 years | 2002 | Suppressed | 39992194 | Suppressed | Suppressed | Suppressed |
| 45-54 years | 2003 | Suppressed | 40819954 | Suppressed | Suppressed | Suppressed |
| 45-54 years | 2004 | Suppressed | 41629930 | Suppressed | Suppressed | Suppressed |
| 45-54 years | 2005 | Suppressed | 42495904 | Suppressed | Suppressed | Suppressed |
| 45-54 years | 2006 | Suppressed | 43286159 | Suppressed | Suppressed | Suppressed |
| 45-54 years | 2007 | Suppressed | 43939939 | Suppressed | Suppressed | Suppressed |
| 45-54 years | 2008 | Suppressed | 44460447 | Suppressed | Suppressed | Suppressed |
| 45-54 years | 2009 | Suppressed | 44867088 | Suppressed | Suppressed | Suppressed |
| 45-54 years | 2010 | Suppressed | 45006716 | Suppressed | Suppressed | Suppressed |
| 45-54 years | 2011 | Suppressed | 44718203 | Suppressed | Suppressed | Suppressed |
| 45-54 years | 2012 | Suppressed | 44268738 | Suppressed | Suppressed | Suppressed |
| 45-54 years | 2013 | Suppressed | 43767532 | Suppressed | Suppressed | Suppressed |
| 45-54 years | 2014 | 0 | 43458851 | Unreliable | 0 | 0 |
| 45-54 years | 2015 | Suppressed | 43188161 | Suppressed | Suppressed | Suppressed |
| 45-54 years | 2016 | Suppressed | 42786679 | Suppressed | Suppressed | Suppressed |
| 45-54 years | 2017 | Suppressed | 42374952 | Suppressed | Suppressed | Suppressed |
| 45-54 years | 2018 | Suppressed | 41631699 | Suppressed | Suppressed | Suppressed |
| 45-54 years | 2019 | Suppressed | 40874902 | Suppressed | Suppressed | Suppressed |
| 45-54 years | 2020 | Suppressed | 40366133 | Suppressed | Suppressed | Suppressed |
| 45-54 years | 2021 | Suppressed | 40688436 | Suppressed | Suppressed | Suppressed |
| 45-54 years | 2022 | 10 | 40431645 | 0.025 | 0.012 | 0.045 |
| 45-54 years | 2023 | Suppressed | 40493781 | Suppressed | Suppressed | Suppressed |
| 45-54 years | 2024 | Suppressed | 40780356 | Suppressed | Suppressed | Suppressed |
| 55-64 years | 1999 | Suppressed | 23778026 | Suppressed | Suppressed | Suppressed |
| 55-64 years | 2000 | 12 | 24274684 | Unreliable | 0.026 | 0.086 |
| 55-64 years | 2001 | 12 | 25105295 | Unreliable | 0.025 | 0.083 |
| 55-64 years | 2002 | 17 | 26703332 | Unreliable | 0.037 | 0.102 |
| 55-64 years | 2003 | 14 | 28008945 | Unreliable | 0.027 | 0.084 |
| 55-64 years | 2004 | 17 | 29305304 | Unreliable | 0.034 | 0.093 |
| 55-64 years | 2005 | 11 | 30641497 | Unreliable | 0.018 | 0.064 |
| 55-64 years | 2006 | Suppressed | 31930113 | Suppressed | Suppressed | Suppressed |
| 55-64 years | 2007 | 12 | 33128434 | Unreliable | 0.019 | 0.063 |
| 55-64 years | 2008 | 10 | 34157063 | Unreliable | 0.014 | 0.054 |
| 55-64 years | 2009 | 14 | 35405600 | Unreliable | 0.022 | 0.066 |
| 55-64 years | 2010 | Suppressed | 36482729 | Suppressed | Suppressed | Suppressed |
| 55-64 years | 2011 | 11 | 38062140 | Unreliable | 0.014 | 0.052 |
| 55-64 years | 2012 | Suppressed | 38586202 | Suppressed | Suppressed | Suppressed |
| 55-64 years | 2013 | 13 | 39316431 | Unreliable | 0.018 | 0.057 |
| 55-64 years | 2014 | 13 | 40077581 | Unreliable | 0.017 | 0.055 |
| 55-64 years | 2015 | 19 | 40877819 | Unreliable | 0.028 | 0.073 |
| 55-64 years | 2016 | 16 | 41463144 | Unreliable | 0.022 | 0.063 |
| 55-64 years | 2017 | 12 | 41995658 | Unreliable | 0.015 | 0.05 |
| 55-64 years | 2018 | 22 | 42272636 | 0.052 | 0.033 | 0.079 |
| 55-64 years | 2019 | 16 | 42448537 | Unreliable | 0.022 | 0.061 |
| 55-64 years | 2020 | 14 | 42403677 | Unreliable | 0.018 | 0.055 |
| 55-64 years | 2021 | 18 | 42803064 | 0.042 | 0.025 | 0.066 |
| 55-64 years | 2022 | 10 | 42085437 | 0.024 | 0.011 | 0.044 |
| 55-64 years | 2023 | 19 | 41854411 | 0.045 | 0.027 | 0.071 |
| 55-64 years | 2024 | 25 | 41661725 | 0.06 | 0.039 | 0.089 |
| 65-74 years | 1999 | 39 | 18418909 | 0.212 | 0.151 | 0.289 |
| 65-74 years | 2000 | 43 | 18390986 | 0.234 | 0.169 | 0.315 |
| 65-74 years | 2001 | 34 | 18384179 | 0.185 | 0.128 | 0.258 |
| 65-74 years | 2002 | 33 | 18388535 | 0.179 | 0.124 | 0.252 |
| 65-74 years | 2003 | 46 | 18500915 | 0.249 | 0.182 | 0.332 |
| 65-74 years | 2004 | 28 | 18667533 | 0.15 | 0.1 | 0.217 |
| 65-74 years | 2005 | 18 | 18881697 | Unreliable | 0.056 | 0.151 |
| 65-74 years | 2006 | 23 | 19203027 | 0.12 | 0.076 | 0.18 |
| 65-74 years | 2007 | 30 | 19698727 | 0.152 | 0.103 | 0.217 |
| 65-74 years | 2008 | 27 | 20505679 | 0.132 | 0.087 | 0.192 |
| 65-74 years | 2009 | 30 | 21233099 | 0.141 | 0.095 | 0.202 |
| 65-74 years | 2010 | 23 | 21713429 | 0.106 | 0.067 | 0.159 |
| 65-74 years | 2011 | 31 | 22481738 | 0.138 | 0.094 | 0.196 |
| 65-74 years | 2012 | 22 | 23985392 | 0.092 | 0.057 | 0.139 |
| 65-74 years | 2013 | 22 | 25216766 | 0.087 | 0.055 | 0.132 |
| 65-74 years | 2014 | 27 | 26398290 | 0.102 | 0.067 | 0.149 |
| 65-74 years | 2015 | 39 | 27550517 | 0.142 | 0.101 | 0.194 |
| 65-74 years | 2016 | 32 | 28630330 | 0.112 | 0.076 | 0.158 |
| 65-74 years | 2017 | 39 | 29683446 | 0.131 | 0.093 | 0.18 |
| 65-74 years | 2018 | 41 | 30492316 | 0.134 | 0.096 | 0.182 |
| 65-74 years | 2019 | 36 | 31483433 | 0.114 | 0.08 | 0.158 |
| 65-74 years | 2020 | 50 | 32549398 | 0.154 | 0.114 | 0.203 |
| 65-74 years | 2021 | 53 | 33666122 | 0.157 | 0.118 | 0.206 |
| 65-74 years | 2022 | 42 | 33788439 | 0.124 | 0.09 | 0.168 |
| 65-74 years | 2023 | 67 | 34685284 | 0.193 | 0.15 | 0.245 |
| 65-74 years | 2024 | 54 | 35444962 | 0.152 | 0.114 | 0.199 |
| 75-84 years | 1999 | 106 | 12224914 | 0.867 | 0.702 | 1.032 |
| 75-84 years | 2000 | 93 | 12361180 | 0.752 | 0.607 | 0.922 |
| 75-84 years | 2001 | 109 | 12593618 | 0.866 | 0.703 | 1.028 |
| 75-84 years | 2002 | 106 | 12764864 | 0.83 | 0.672 | 0.988 |
| 75-84 years | 2003 | 98 | 12896438 | 0.76 | 0.617 | 0.926 |
| 75-84 years | 2004 | 107 | 12989903 | 0.824 | 0.668 | 0.98 |
| 75-84 years | 2005 | 90 | 13074802 | 0.688 | 0.554 | 0.846 |
| 75-84 years | 2006 | 93 | 13095151 | 0.71 | 0.573 | 0.87 |
| 75-84 years | 2007 | 59 | 13087439 | 0.451 | 0.343 | 0.582 |
| 75-84 years | 2008 | 63 | 13076102 | 0.482 | 0.37 | 0.616 |
| 75-84 years | 2009 | 58 | 13022775 | 0.445 | 0.338 | 0.576 |
| 75-84 years | 2010 | 56 | 13061122 | 0.429 | 0.324 | 0.557 |
| 75-84 years | 2011 | 59 | 13175230 | 0.448 | 0.341 | 0.578 |
| 75-84 years | 2012 | 57 | 13272634 | 0.429 | 0.325 | 0.556 |
| 75-84 years | 2013 | 52 | 13446519 | 0.387 | 0.289 | 0.507 |
| 75-84 years | 2014 | 49 | 13682690 | 0.358 | 0.265 | 0.473 |
| 75-84 years | 2015 | 59 | 13923174 | 0.424 | 0.323 | 0.547 |
| 75-84 years | 2016 | 69 | 14233534 | 0.485 | 0.377 | 0.614 |
| 75-84 years | 2017 | 56 | 14706551 | 0.381 | 0.288 | 0.494 |
| 75-84 years | 2018 | 71 | 15394374 | 0.461 | 0.36 | 0.582 |
| 75-84 years | 2019 | 70 | 15969872 | 0.438 | 0.342 | 0.554 |
| 75-84 years | 2020 | 89 | 16451547 | 0.541 | 0.434 | 0.666 |
| 75-84 years | 2021 | 97 | 16206075 | 0.599 | 0.485 | 0.73 |
| 75-84 years | 2022 | 98 | 17520545 | 0.559 | 0.454 | 0.682 |
| 75-84 years | 2023 | 115 | 18368097 | 0.626 | 0.512 | 0.741 |
| 75-84 years | 2024 | 130 | 19299813 | 0.674 | 0.558 | 0.789 |
| 85+ years | 1999 | 197 | 4154018 | 4.742 | 4.08 | 5.405 |
| 85+ years | 2000 | 194 | 4239587 | 4.576 | 3.932 | 5.22 |
| 85+ years | 2001 | 188 | 4312494 | 4.359 | 3.736 | 4.983 |
| 85+ years | 2002 | 183 | 4368808 | 4.189 | 3.582 | 4.796 |
| 85+ years | 2003 | 188 | 4466176 | 4.209 | 3.608 | 4.811 |
| 85+ years | 2004 | 182 | 4545883 | 4.004 | 3.422 | 4.585 |
| 85+ years | 2005 | 195 | 4693299 | 4.155 | 3.572 | 4.738 |
| 85+ years | 2006 | 213 | 4865929 | 4.377 | 3.79 | 4.965 |
| 85+ years | 2007 | 158 | 5039545 | 3.135 | 2.646 | 3.624 |
| 85+ years | 2008 | 169 | 5195840 | 3.253 | 2.762 | 3.743 |
| 85+ years | 2009 | 168 | 5367301 | 3.13 | 2.657 | 3.603 |
| 85+ years | 2010 | 162 | 5493433 | 2.949 | 2.495 | 3.403 |
| 85+ years | 2011 | 166 | 5737173 | 2.893 | 2.453 | 3.334 |
| 85+ years | 2012 | 146 | 5887330 | 2.48 | 2.078 | 2.882 |
| 85+ years | 2013 | 176 | 6040789 | 2.914 | 2.483 | 3.344 |
| 85+ years | 2014 | 124 | 6162231 | 2.012 | 1.658 | 2.366 |
| 85+ years | 2015 | 157 | 6287161 | 2.497 | 2.107 | 2.888 |
| 85+ years | 2016 | 186 | 6380331 | 2.915 | 2.496 | 3.334 |
| 85+ years | 2017 | 187 | 6468682 | 2.891 | 2.477 | 3.305 |
| 85+ years | 2018 | 187 | 6544503 | 2.857 | 2.448 | 3.267 |
| 85+ years | 2019 | 207 | 6604958 | 3.134 | 2.707 | 3.561 |
| 85+ years | 2020 | 188 | 6658420 | 2.823 | 2.42 | 3.227 |
| 85+ years | 2021 | 212 | 5975756 | 3.548 | 3.07 | 4.025 |
| 85+ years | 2022 | 225 | 6485868 | 3.469 | 3.016 | 3.922 |
| 85+ years | 2023 | 236 | 6194980 | 3.81 | 3.323 | 4.296 |
| 85+ years | 2024 | 279 | 6435143 | 4.336 | 3.827 | 4.844 |

**Table S6:** Age Group Stratified Heart Failure Related Mortality among Breast Cancer Patients in the United States, 1999-2024.

| **Year** | **Medical Facility** | | | | **Decedent's home** | **Hospice Facility** | **Nursing home/long term care** | **Other** | **Place of death unknown** |
| --- | --- | --- | --- | --- | --- | --- | --- | --- | --- |
|  | **Inpatient** | **Outpatient or ER** | **Dead on Arrival** | **Status unknown** |  |  |  |  |  |
| 1999 | 104 | 11 | Suppressed | Suppressed | 84 | Missing | 142 | Suppressed | 0 |
| 2000 | 99 | 14 | Suppressed | Suppressed | 72 | Missing | 145 | 12 | 0 |
| 2001 | 125 | 13 | Suppressed | Suppressed | 69 | Missing | 125 | 11 | 0 |
| 2002 | 95 | 24 | Suppressed | 0 | 79 | Missing | 135 | 11 | 0 |
| 2003 | 101 | 11 | Suppressed | Missing | 89 | Suppressed | 125 | 18 | 0 |
| 2004 | 113 | 18 | Suppressed | Missing | 74 | 0 | 122 | 10 | 0 |
| 2005 | 85 | 12 | Suppressed | Missing | 71 | Suppressed | 132 | 16 | Suppressed |
| 2006 | 92 | 12 | 0 | Missing | 84 | Suppressed | 131 | 18 | Suppressed |
| 2007 | 67 | Suppressed | Suppressed | Missing | 61 | Suppressed | 103 | 23 | 0 |
| 2008 | 72 | Suppressed | Suppressed | Missing | 79 | Suppressed | 92 | 15 | Suppressed |
| 2009 | 70 | Suppressed | 0 | Missing | 69 | Suppressed | 100 | 12 | Suppressed |
| 2010 | 52 | 18 | Suppressed | Missing | 62 | Suppressed | 91 | 18 | Suppressed |
| 2011 | 62 | Suppressed | Suppressed | Missing | 72 | 14 | 98 | 12 | 0 |
| 2012 | 45 | Suppressed | Suppressed | Missing | 79 | 10 | 80 | 10 | 0 |
| 2013 | 64 | Suppressed | 0 | Missing | 93 | 17 | 76 | 16 | 0 |
| 2014 | 48 | 11 | 0 | Missing | 72 | Suppressed | 66 | Suppressed | 0 |
| 2015 | 55 | 12 | 0 | Missing | 96 | 16 | 84 | 15 | 0 |
| 2016 | 75 | 17 | 0 | Missing | 103 | 15 | 82 | 16 | 0 |
| 2017 | 56 | 17 | 0 | Missing | 97 | 22 | 95 | 13 | 0 |
| 2018 | 65 | Suppressed | 0 | Missing | 111 | 28 | 101 | 12 | 0 |
| 2019 | 65 | 10 | 0 | Missing | 110 | 20 | 107 | 21 | Suppressed |
| 2020 | 58 | 14 | 0 | Missing | 131 | 36 | 91 | 15 | 0 |
| 2021 | 76 | 22 | Suppressed | 0 | 139 | 28 | 93 | 26 | Suppressed |
| 2022 | 82 | 15 | Suppressed | 0 | 144 | 28 | 96 | 19 | 0 |
| 2023 | 101 | 16 | 0 | 0 | 156 | 32 | 114 | 25 | 0 |
| 2024 | 97 | 20 | Suppressed | 0 | 183 | 38 | 125 | 30 | 0 |

**Table S7:** Total deaths due to Heart Failure in Breast Cancer Patients stratified by Place of Death in the United States, 1999-2024.
